# Protein kinase D1 in myeloid lineage cells contributes to the accumulation of CXCR3^+^CCR6^+^ nonconventional Th1 cells in the lungs and potentiates hypersensitivity pneumonitis caused by *S. rectivirgula*

**DOI:** 10.3389/fimmu.2024.1403155

**Published:** 2024-10-11

**Authors:** John D. Snyder, Tae Won Yoon, Sangmin Lee, Priyanka Halder, Elizabeth Ann Fitzpatrick, Ae-Kyung Yi

**Affiliations:** ^1^ Integrated Biomedical Science Graduate Program, The University of Tennessee Health Science Center, Memphis, TN, United States; ^2^ Department of Microbiology, Immunology and Biochemistry, The University of Tennessee Health Science Center, Memphis, TN, United States

**Keywords:** protein kinase D1, cytokines/chemokines, inflammation, alveolitis, *Saccharopolyspora rectivirgula*, hypersensitivity pneumonitis, toll-like receptor signaling

## Abstract

**Introduction:**

Hypersensitivity pneumonitis (HP) is an extrinsic allergic alveolitis characterized by inflammation of the interstitium, bronchioles, and alveoli of the lung that leads to granuloma formation. We previously found that activation of protein kinase D1 (PKD1) in the lungs following exposures to *Saccharopolyspora rectivirgula* contributes to the acute pulmonary inflammation, IL-17A expression in the lungs, and development of HP. In the present study, we investigated whether PKD1 in myeloid-lineage cells affects the pathogenic course of the *S. rectivirgula*-induced HP.

**Methods:**

Mice were exposed intranasally to *S. rectivirgula* once or 3 times/week for 3 weeks. The protein and mRNA expression levels of cytokines/chemokines were detected by enzyme-linked immunosorbent assay and quantitative real-time PCR, respectively. Flow cytometry was used to detect the different types of immune cells and the levels of surface proteins. Lung tissue sections were stained with hematoxylin and eosin, digital images were captured, and immune cells influx into the interstitial lung tissue were detected.

**Results:**

Compared to control PKD1-sufficient mice, mice with PKD1 deficiency in myeloid-lineage cells (PKD1mKO) showed significantly suppressed expression of TNFα, IFNγ, IL-6, CCL2, CCL3, CCL4, CXCL1, CXCL2, and CXCL10 and neutrophilic alveolitis after single intranasal exposure to *S. rectivirgula*. Substantially reduced levels of alveolitis and granuloma formation were observed in the PKD1mKO mice repeatedly exposed to *S. rectivirgula* for 3 weeks. In addition, expression levels of the Th1/Th17 polarizing cytokines and chemokines such as IFNγ, IL-17A, CXCL9, CXCL10, CXCL11, and CCL20 in lungs were significantly reduced in the PKD1mKO mice repeatedly exposed to *S. rectivirgula*. Moreover, accumulation of CXCR3+CCR6+ nonconventional Th1 in the lungs were significantly reduced in PKD1mKO mice repeatedly exposed to *S. rectivirgula*.

**Discussion:**

Our results demonstrate that PKD1 in myeloid-lineage cells plays an essential role in the development and progress of HP caused by repeated exposure to *S. rectivirgula* by contributing Th1/Th17 polarizing proinflammatory responses, alveolitis, and accumulation of pathogenic nonconventional Th1 cells in the lungs.

## Introduction

Hypersensitivity pneumonitis (HP), also known as an extrinsic allergic alveolitis, is an immune-mediated inflammatory and granulomatous interstitial lung disease (ILD) characterized by a neutrophilic/lymphocytic inflammation of the interstitium, bronchioles, and alveoli and granuloma formation in the lung, and, in some patients, progresses to irreversible fibrosis and end-stage lung damage with respiratory failure (1, 2). HP is caused by intense or repeated inhalation of antigenic organic particles or low-molecular-weight chemicals in a variety of environmental settings in genetically susceptible individuals (1, 2). Numerous organic antigens (over 300 antigens) broadly classified into bacteria, fungi, animal proteins, plant proteins, wood dusts, low-molecular-weight chemicals, and metals have been described to cause HP (3). These antigens are often encountered in the daily life of patients suffering from disease; thus, avoiding exposure is challenging. HP is often related to a person’s occupational environments as reflected in its other names (e.g., bird fancier’s lung, famer’s lung, or hot tub lung). *Saccharopolyspora rectivirgula*, a thermophilic actinomycetes commonly found in moldy hay, is one of the HP-inciting antigens that cause farmer’s lung disease (one of the most common types of HP seen in agricultural workers) (4). HP may present as acute, subacute, or chronic illness with unique clinical presentations (5). Following inhalation of HP-inciting agents, patients develop severe neutrophilic inflammation in the lung. Continued antigen exposures result in a positive feedback loop, leading to increased production of interleukin (IL)-1, IL-6, IL-12, and tumor necrosis factor α (TNFα) by alveolar macrophages and increased production of interferon γ (IFNγ) by activated neutrophils and Th1 cells (6–9). Increased levels of L-selectins in macrophages and neutrophils, intracellular adhesion molecule-1 (ICAM-1), CD80 and CD86 in macrophages, CD28 in lymphocytes, and E-selectin in endothelial cells in lung tissues of HP patients correlate with the lymphocytosis and the persistence of inflammation (10–13). As the disease progresses to the subacute form, the alveolitis becomes progressively more lymphocytic, and granulomas develop in the interstitial spaces. Lymphocytes can account for 60%–90% of bronchoalveolar lavage (BAL)-recovered cells from HP patients (14, 15). Several chemokines, including CC motif chemokine ligand (CCL) 2, CCL18, CXC motif chemokine ligand (CXCL) 8, and CXCL9 are increased in the BAL fluid (BALF) of HP patients and contribute to the development of the cellular infiltrate and granulomas in HP (16–20). The chronic phase of HP can be non-fibrotic or fibrotic, and patents with fibrosis have worse prognosis and a higher risk for morbidity and mortality (21–23).

The pathogenic mechanisms involved in initiation and progression of HP are complex and remain incompletely understood. The intricate interplays between the host genetic factors, the host immune system, the environment, and the causative agents affect the disease pathogenesis. Toll-like receptors (TLRs) on/in innate immune cells recognize specific molecular patterns present in microbial pathogens, foreign particulates, and injured or altered self-tissues, and then initiate and amplify cascades of inflammatory responses (24–31). Increased expression of TLR2, TLR4, TLR6, and TLR8 in mice with HP has been reported (32, 33). Using animal models of HP, previous studies have demonstrated that TLR2, TLR4, TLR6, and TLR9 are involved in the detection of numerous HP-inciting agents and are critical contributors to lung inflammation and HP pathogenesis (34–40). TLR2, TLR6, and TLR9 have been demonstrated to be involved in mediating proinflammatory responses, neutrophil recruitment to airway, and Th17 development after acute and chronic exposures to an HP-inciting antigen *S. rectivirgula* (34, 35, 39). Previous studies also showed that the initial expression of the majority of proinflammatory mediators and neutrophil infiltration into the lung after exposure to HP-inciting agents are largely dependent on myeloid differentiation factor 88 (MyD88), the major signaling adaptor in the TLR pathway (38, 41–44). In addition, the receptors of IL-1 family cytokines (such as IL-1 and IL-18 that have been shown to be increased in HP patients and contribute to enhanced Th17-type responses) share their signal transduction pathway with TLRs by utilizing MyD88 as the signaling adaptor (7, 45, 46). These findings support the concept that, although there is a broad spectrum of agents that cause HP, interruption of the MyD88-dependent signaling pathway might have significant beneficial effects on attenuation of the disease progress and the signaling modulators downstream of MyD88 would be a reasonable therapeutic target for HP.

The protein kinase D1 (PKD1) is a member of the PKD family, which is composed of three structurally related serine/threonine kinases: PKD1 (PKCμ), PKD2, and PKD3 (PKCν) (47). Several previous studies have found a link between PKD1 and TLR/IL-1R signaling. TLR4-mediated p38 activation and TNFα secretion in microglial cells, TLR5-mediated p38 activation and CXCL8 production in epithelial cells, and TLR1/2-induced expression of heat shock protein 27 and CCL2 in mast cells are suppressed by Gö6976, a pharmacological inhibitor that inhibits the activity of protein kinase Cα (PKCα), PKCβ, checkpoint kinases 1 and 2 (CHK1/2), PKD1, PKD2, and PKD3 (48–52). We also found that PKD1, but not PKD2 and PKD3, is recruited to the MyD88/IL-1R-associated kinase (IRAK)/TNFR-associated factor 6 (TRAF6) signaling complex and becomes activated upon stimulation by TLR ligands (TLR1, TLR2, TLR4, TLR5, TLR6, TLR7, TLR8, or TLR9), IL-1β, IL-18, Group B streptococci, or *S. rectivirgula*. This PKD1 activation by TLR/IL-1R is required for the MyD88-dependent ubiquitination of TRAF6 and activation of TGFβ-activated kinase 1 (TAK1), NF-κB, and mitogen-activated protein kinases (MAPKs), leading to the subsequent expression of proinflammatory mediators such as TNFα, IL-6, IL-12, chemokine CCL2, and costimulatory molecule CD86 (43, 53–56).

Previously, we found that *S. rectivirgula* induces activation of PKD1 in an MyD88-dependent manner in the mouse lung *in vivo* and in murine cell lines MLE12 (alveolar type II epithelial cells), MPRO (promyelocytes differentiated to neutrophils), and AMJ2-C11 (alveolar macrophages) (43). *S. rectivirgula*-mediated cytokine and chemokine production in these cell lines are significantly inhibited when PKD1 expression is knocked down by PKD1-specific siRNA or when PKD1 enzyme activity is inhibited by Gö6976 (43). *S. rectivirgula*-induced acute lung injury accompanied by neutrophilic alveolitis and increased production of proinflammatory cytokines TNFα, IL-6, IL-12, and IFNγ and chemokines CCL2 and CXCL2 in the lungs are also significantly suppressed in mice pre-treated with PKD inhibitor Gö6976 or in PKD1-insufficient mice (PKD1KO) (43, 57). In addition, PKD1-insufficient mice (compared to wild-type PKD1-sufficient mice) showed significantly reduced levels of alveolitis, surface MHC-II expression on macrophages and neutrophils infiltrated into airways and interstitial lung spaces, expression of IFNγ, IL-17A, and CXCL9 in the lungs, and granuloma formation following 5-week repeated exposures to *S. rectivirgula* (57). These findings imply that PKD1 is one of the critical factors required for acute and chronic pulmonary proinflammatory responses that contribute to T helper (Th) 1- and Th17-promoting milieu in the lungs and development of HP. Myeloid lineage cells, particularly macrophages and neutrophils, are crucial in the early stages of lung inflammation in HP (58, 59). They highly express TLRs, detect the presence of inciting agents, release inflammatory cytokines/chemokines to activate and attract more immune cells to the lungs, influence Th cell lineage differentiation, and express MHC-II and costimulatory molecules to activate T cells and prime the immune system towards its adaptive response (10, 60–63). In the present study, we investigated whether PKD1 in myeloid cells plays an essential role in mediating acute lung injury after exposure to *S. rectivirgula*. Additionally, we investigated whether PKD1 in myeloid cells contributes to the neutrophilic/lymphocytic alveolitis and the cytokine/chemokine milieu that affects the pulmonary accumulation of pathogenic Th1 and Th17 cells during the development of HP caused by repeated exposure to *S. rectivirgula*.

## Materials and methods

### Mice

C57BL/6 mice and Lyz*^Cre^* mice (B6.129P2-*Lyz2^tm1(cre)lfo^*/J, JAX stock #018956) were purchased from The Jackson Laboratory (Bar Harbor, ME). The generation and characterization of Lyz*^Cre^* mice (express Cre in myeloid cells due to targeted insertion of the *cre* cDNA into their endogenous M lysozyme locus) were reported by Förster and colleagues (64). PKD1*^fl/fl^* mice generated as previously described (65) were kindly provided by Dr. E. Olson (University of Texas Southwestern Medical Center, Dallas, TX). PKD1*^fl/fl^* mice and Lyz*^Cre^* mice were backcrossed onto the C57BL/6 more than seven generations at the University of Tennessee Health Science Center (UTHSC). Subsequent cross-breeding of these PKD1*^fl/fl^* and Lyz*^Cre^* mice resulted in PKD1*^fl/fl^*-Lyz*^Cre^* (myeloid lineage cell-specific PKD1-deficient, PKD1mKO) mice. PKD1*^fl/fl^* mice were used as wild-type (WT) control and PKD1*^fl/fl^*-Lyz*^Cre+/-^* mice were used as PKD1mKO. Mice were bred and maintained in a pathogen-free facility at UTHSC. All animal care and housing requirements set forth by the National Institutes of Health Committee on Care and Use of Laboratory Animals of the Institute of Laboratory Animal Resources were followed. Animal protocols were reviewed and approved by the UTHSC Institutional Animal Care and Use Committee.

### *Saccharopolyspora rectivirgula* preparation and challenge, bronchoalveolar lavage, lung interstitial cell isolation, and determination of alveolitis

*S. rectivirgula* (ATCC^®^15347™) was prepared as previously described (57). Age- and gender-matched mice were exposed intranasally to *S. rectivirgula* (80 μg/mouse or 100 μg/mouse) or endotoxin-free saline (AdipoGen Life Sciences, San Diego, CA) for one time or three times per week for 3 weeks. Mice were euthanized at designated time points (2, 6, 24, 48, or 72 h after the last *S. rectivirgula* challenge). Unless otherwise indicated, control and *S. rectivirgula*-exposed mice were analyzed individually. To perform BAL, mice tracheas were cannulated, and the lungs were washed with 1 mL of 2 mM EDTA/PBS. The typical amount of fluid recovered was approximately 70% of the input. The recovered BALF was centrifuged, and the resulting supernatants were kept at −80°C until used for detection of cytokines and chemokines. The cells recovered from the BALF were used to determine the degree of alveolitis by counting the number of live cells using a hemocytometer or Countess II (Invitrogen) following trypan blue staining. The cellular composition of the alveolitis was determined by flow cytometric analysis. To isolate lung interstitial cells (LICs), the lungs were perfused through the right ventricle with PBS and digested with collagenase (20 U/mL, type 4, LS004186, Worthington Biochemical Co, Lakewood, NJ) and deoxyribonuclease I (40 μg/mL, LS002004, Worthington Biochemical Co) for 45 min at 37°C. Cells were freed by mechanical disruption and filtered through 40 μM nylon mesh. Discontinuous Percoll (P4937, Sigma-Aldrich, St. Louis, MO) gradient centrifugation was used to separate extraneous fibroblasts or epithelial cells. The mononuclear cells were isolated at the 40/80% interface. The number of live cells were counted using a hemocytometer or Countess II (Invitrogen) following trypan blue staining and used for flow cytometric analysis.

### Isolation of spleen cells, peritoneal macrophages, thymocytes, and bone marrow neutrophils

Spleen cells were obtained as previously described (54). To isolate peritoneal macrophages, a peritoneal lavage was performed by injecting 8 mL of cold sterile PBS (Corning, Manassas, VA) into the peritoneal cavity of mice followed by a gentle massage on the peritoneal cavity and subsequent aspiration of the peritoneal fluid. Peritoneal cells were obtained from the recovered peritoneal fluid by centrifugation. After removing RBC using RBC lysis buffer, peritoneal cells were washed three times with PBS, suspended in complete medium (RPMI 1640 supplemented with 100 IU/mL penicillin, 100 μg/mL streptomycin, and 10% heat-inactivated FBS), placed in plastic petri dishes, and then incubated for 1 h at 37°C. Suspended cells were removed. Plastic adherent cells were washed three times with prewarmed PBS and then collected by scraping. The resulting plastic adherent cells were used as peritoneal macrophages for confirming PKD1 gene deletion in myeloid lineage cells in PKD1mKO. To isolate thymocytes, thymus lobes from mice were harvested as described previously (66). Thymus cells were obtained by cutting thymus lobes into small pieces with fine scissors, crushed with a syringe plunger in cold PBS, and then filtered through a 100-μm sterile cell strainer (Fisher Scientific). Thymic cells in suspensions were washed three times with PBS and then used as thymocytes. To isolate neutrophils from bone marrow, bone marrow cells from mice were prepared as previously described (55). After removing RBC, the bone marrow cells were washed three times with PBS and then resuspended in PBS. The bone marrow cell suspension was overlaid on top of the Ficoll-Paque Plus (GE Healthcare, Uppsala, Sweden) and then centrifuged for 30 min at room temperature without break. After discarding upper layers, granulocytes pelleted in the bottom were washed three times with PBS and then used as neutrophils. Purity of isolated neutrophils were analyzed by Diff-Quick staining (Diff Quick Stain kit, IMEB Inc., San Marcos, CA) according to the manufacturer’s instructions and approximately 85% to 90% of the isolated cells were morphologically neutrophils/granulocytes. RPMI 1640 was purchased from Corning, Pen Strep was purchased from Gibco, and FBS was purchased from R&D Systems (Flowery Branch, GA).

### Trans-well neutrophil migration assay

To evaluate the migration abilities of neutrophils, 1 mL of RPMI-1640 media or murine recombinant CXCL2 (50 ng/mL, PeproTech, cat# 250-15) was placed into the bottom chamber of each designated well in a 24-well plate and 5 × 10^5^ bone marrow neutrophils (isolated from WT or PKD1mKO) in 200 µL of RPMI-1640 media were placed into the top chamber (Greiner Bio-One, ThinCert 3.0 μm pore, 24-well. ref#662-631). The trans-well plate was incubated at 37°C with 5% CO_2_ for 2 h and then neutrophils that migrated into the bottom chamber and the bottom chamber side of the membrane were collected and counted using Countess II (Invitrogen).

### Genomic DNA isolation, genotyping, total RNA isolation, reverse transcription polymerase chain reaction, and real-time quantitative polymerase chain reaction

Genomic DNA was isolated from mouse tail clips using a Phire Tissue Direct PCR Master Mix (ThermoFisher Scientific Inc., Vilnius, Lithuania) following the manufacturer’s protocol. To distinguish PKD1 alleles and lysozyme 2 (Lyz2) alleles, genomic DNA was analyzed by PCR performed in a thermocycler (T100 Thermal Cycler, Bio-Rad). The sequences of PCR primers used for PKD1-WT/loxP and KO were described previously (57). The sequences of PCR primers used for Lyz*^Cre^* are as follows: Mutant F: 5′CCCAGAAATGCCAGATTACG3′, WT F: 5′TTACAGTCGGCCAGGCTGAC3′, and Common R: 5′CTTGGGCTGCCAGAATTTCTC3′. All primers for genotyping were purchased from Integrated DNA Technologies, Inc. (IDT, Coralville, IA). DNA-free total RNA was isolated using Direct-Zol RNA Microprep kits (Zymo Research, Irvine, CA) following the manufacturer’s protocol. The relative amounts of the indicated gene transcripts were analyzed by RT-PCR as described previously (56). Sequences of RT-PCR primers used were described previously (54, 57, 67). All primers for RT-PCR were purchased from IDT. For RT real-time qPCR (SYBR Green Assay), total RNA (250 ng) was reverse transcribed using the High-Capacity cDNA Reverse Transcription Kit (Applied Biosystems). Real-time qPCR was performed on a QuantStudio 5 (Applied Biosystems) using the PowerUp SYBR Green Master Mix (Applied Biosystems). Primers were designed and supplied by IDT. The product size was initially monitored by agarose gel electrophoresis. Melting curves were analyzed to control for specificity of PCR reactions. The data on genes that were differentially expressed were normalized to the expression of the housekeeping gene, GAPDH. The relative units were calculated from a standard curve, plotting three different concentrations against the PCR cycle number at which the measured intensity reaches a fixed value (with a 10-fold increment equivalent to ~3.1 cycles). Fold changes comparing *S. rectivirgula*-treated WT mice and *S. rectivirgula*-treated PKD1mKO to control mice exposed to saline were calculated by comparative quantification algorithm-delta delta Ct method (fold difference = 2^−ΔΔCt^). Primer sequence information for qPCR is either described previously (57) or listed in **Supplementary Table 1**.

### Western blot assay, enzyme-linked immunosorbent assay, and multiplex sandwich immunoassay

Levels of specific proteins in whole cell extracts were analyzed by Western blot assay as described previously (54, 68). Blots developed in enhanced chemiluminescence reagents (GE Healthcare, UK) were either exposed onto x-ray film followed by processing through X-OMAT 2000A processor (Kodak). Blots developed with fluorochrome-conjugated secondary Abs were scanned using ODYSSEY CLx (LI-COR, Lincoln, NE). Actin was used as a loading control for all Western blot assays. Antibodies specific for actin was purchased from Santa Cruz Biotechnology, Inc. (Dallas, TX). Antibodies specific for PKD1 were purchased from Origene (Rockville, MD). Concentrations of IFNγ, TNFα, IL-6, and IL-17A in BALF were analyzed by enzyme-linked immunosorbent assay (ELISA) as previously described (54, 68). All ELISA kits were purchased from Invitrogen. Concentrations of IL-1α, IL-1β, CCL2, CXCL1, and CXCL2 in BALF were analyzed using the multiplex sandwich assay kit (MCYTOMAG-70K, EMD Millipore, Billerica, MA) following the manufacturer’s protocol. The fluorescence was measured with Bio-Plex MAGPIX Multiplex Reader (Bio-Rad, Hercules, CA).

### Proteome profiler mouse cytokine array

The relative levels of 40 cytokines and chemokines in BALF obtained from WT and PKD1mKO exposed to *S. rectivirgula* repeatedly for 3 weeks were evaluated using Proteome Profiler Mouse Cytokine Array Kit (ARY006, R&D Systems) following the manufacturer’s instructions. The ChemiDoc™ Touch Imaging system (Bio-Rad) was used to detect the chemiluminescent signal of the bound cytokine/chemokine, and the Image Lab (Bio-Rad) software was utilized to measure the cytokine/chemokine signal intensity of the digital image. Each spot was adjusted for adjacent background intensity and normalized to a positive control on the membrane.

### Identification of interleukin-17A- and interferonγ-producing CD4^+^ T cells by intracellular staining

LICs (4 × 10^6^ cells/mL) were stimulated with phorbol 12-myristat 13-acetate (PMA, 5 ng/mL) and ionomycin (500 ng/mL) for 2 h. Two hours later, 1 μL of BD GolgiPlug (BD Biosciences, San Diego, CA) was added to the culture and then incubated for four additional hours. Cells were harvested, washed with 5% FBS in PBS, stained with DAPI for 2 min on ice, washed, and then incubated with anti-CD16/32 (2.4G2) in PBS containing 5% FBS for 15 min on ice to block Fc receptors. Cells were stained with anti-βTCR (H57-597) and anti-CD4 (RM4-5) for 30 min on ice. Cells were fixed in 250 μL of BD cytofix/cytoperm solution (BD Biosciences) for 20 min on ice. Cells were washed with BD perm/wash solution (BD Biosciences) and intracellularly stained with anti-IL-17A-APC (TC11-18H10.1) and anti-IFNγ-PE (XMG1.2) for 30 min on ice. Cells were washed again with the 1× BD perm/wash solution and then subjected to flow cytometric analysis. All antibodies were purchased from BioLegend (San Diego, CA). Phorbol 12-myristate 13-acetate (PMA) and ionomycin were purchased from Sigma-Aldrich (St. Louis, MO).

### Flow cytometric analysis

For splenic immune cell profiling, spleen cells were incubated with anti-CD16/CD32 Abs and subsequently stained with fluorochrome-conjugated Abs to CD45 (30-F11), CD19 (1D3), βTCR (H57-597), CD4 (GK1.5), CD8 (53-6.7), CD11b (M1/70), Ly6G (1A8), and CD11c (N418). Cells were analyzed using a Cytek Aurora Cytometer (N7-00003, Cytek Biosciences Inc., Fermont, CA) and FlowJo flow cytometry data analysis software (FlowJo LLC, Ashland, OR). Gating strategies for splenic and bone marrow immune cell profiling are shown in **Supplementary Figures 1A, B**, respectively. Briefly, debris and doublets were gated out. In the single-cell gate, CD45^+^ population was gaited and identified as CD45^+^ cells. In the CD45^+^ cell gate, the CD19^+^ population (B cells), βTCR^+^ population (T cells), βTCR^+^CD4^+^ population (CD4^+^ T cells), βTCR^+^CD8^+^ population (CD8^+^ T cells), CD11b^+^ population (monocytic cells), CD11b^+^LyG6^+^ population (neutrophils), and CD11c^+^ population (dendritic cells) were identified. The frequency of each cell population was expressed as the percentage of CD45^+^ cells.

To detect neutrophils in the airway and lung interstitium after one-time *S. rectivirgula* exposure, BAL cells were incubated with anti-CD16/CD32 Abs, subsequently stained with fluorochrome-conjugated Abs to CD11b (M1/70), F4/80 (BM8), and Gr-1 (RB6-8C5), and then analyzed using a BD Biosciences LSR II flow cytometer (BD Biosciences, San Diego, CA) and FlowJo flow cytometry data analysis software. The gating strategy for flow cytometric analysis of neutrophils in BAL cells obtained from mice 24 h after the single *S. rectivirgula* exposure is shown in **Supplementary Figure 2**. Debris and doublets were gated out. In the single-cell gate, the CD11b^+^Gr1^+^ population was gated, and then the Gr1^+^F4/80^-^ population in the CD11b^+^Gr1^+^ gate was identified as neutrophils (CD11b^+^Gr1^+^F4/80^-^). The frequency of CD11b^+^Gr1^+^F4/80^-^ cells was expressed as % of BAL cells.

To determine the cellular composition and levels of certain surface markers of cells recovered from BALF and lung interstitium from mice exposed to *S. rectivirgula* repeatedly for 3 weeks, cells were incubated with anti-CD16/CD32 Abs; subsequently stained with fluorochrome-conjugated Abs to CD45 (I3/2.3), CD11b (M1/70), CD11c (N418), F4/80 (BM8), Gr-1 (RB6-8C5), MHCII (I-A/I-E) (M5/114.15.2), βTCR (H57-597), CD4 (RM4-5), CD8 (53-6.7), CD86 (GL-1), CD206 (C068C2), CD40 (3/23), CCR6 (29-2L17), CXCR3 (CXCR3-173), and/or CD69 (H1.2F3); and then analyzed using a BD Biosciences LSR II flow cytometer or Cytek Aurora (Cytek Biosciences) and FlowJo flow cytometry data analysis software. Gating strategies for flow cytometric analysis of BAL cells and LICs are shown in **Supplementary Figures 3**, **4**, respectively. Debris and doublets were gated out. In the single-cell gate, the CD45^+^ population was identified. For myeloid lineage cells, the CD11b^-^CD11c^-^ population was gated out in the CD45^+^ population. In the rest of CD45^+^ cells, the CD11c^+^F4/80^+^ population was identified as alveolar macrophages (AM), and the remaining cells were identified as the non-AM population. The non-AM population was divided into the CD11b^+^Gr1^+^ population and the non-polymorphonuclear neutrophils (PMN) population. In the CD11b^+^Gr1^+^ population, the CD11b^+^Gr1^+^F4/80^-^ population was identified as neutrophils. In the non-PMN population, the CD11b^+^F4/80^+^ population was identified as macrophages (MAC), and the F4/80^-^ population was identified as the non-MAC population. In the non-MAC population, the CD11c^+^CD11b^-^F4/80^-^ population and CD11c^+^CD11b^+^F4/80^-^ population were identified. In the non-MAC population, the CD11c^+^CD11b^-^F4/80^-^MHC-II^+^ population was identified as conventional dendritic cell type 1 (cDC1) and the CD11c^+^CD11b^+^F4/80^-^MHC-II^+^ population was identified as conventional dendritic cell type 2 (cDC2). For T cells, βTCR^+^ cells were identified in the CD45^+^ population and then the CD4^+^βTCR^+^ population was identified as CD4^+^ T cells, and the CD8^+^βTCR^+^ population was identified as CD8^+^ T cells. The frequency of each cell population was expressed as % of CD45^+^ cells. The surface expression levels of various markers (CD86, MHC-II, CD40, CD206, CD69, CXCR3, and CCR6) on cells were determined by their geometric mean fluorescent intensity (gMFI). The gMFI of each marker was calculated for each indicated cell population using the FlowJo flow cytometry data analysis software. All Abs were purchased from BioLegend (San Diego, CA), eBioscience, TONBO, or BD Pharmingen.

### Lung histology

The left lung lobes removed from the mice were fixed in 10% neutral buffered formalin, dehydrated, and embedded in paraffin. The paraffin-embedded lungs were sectioned longitudinally at 5 μm and stained with hematoxylin and eosin (H&E). To determine inflammatory cell influx into the interstitial lung tissue, digital images of whole H&E-stained slides were captured using the Aperio ScanScope^®^XT Slide Scanner (Aperio Technologies, Inc. Vista CA).

### Statistical analysis

Data were expressed as mean values ± SD. The differences between two groups were evaluated using two-tailed Student’s *t*-test. The differences between multiple groups were evaluated using one-way ANOVA with Tukey’s *post-hoc* test. GraphPad Prism statistical software (GraphPad Software, San Diego, CA) was used. Statistical differences with *p* < 0.05, *p* < 0.01, *p* < 0.001, and *p* < 0.0001 are indicated in the figures as *, **, ***, and ****, respectively, and considered significant.

## Results

### Conditional protein kinase D1 deletion in myeloid lineage cells in mice

**Figure 1:**
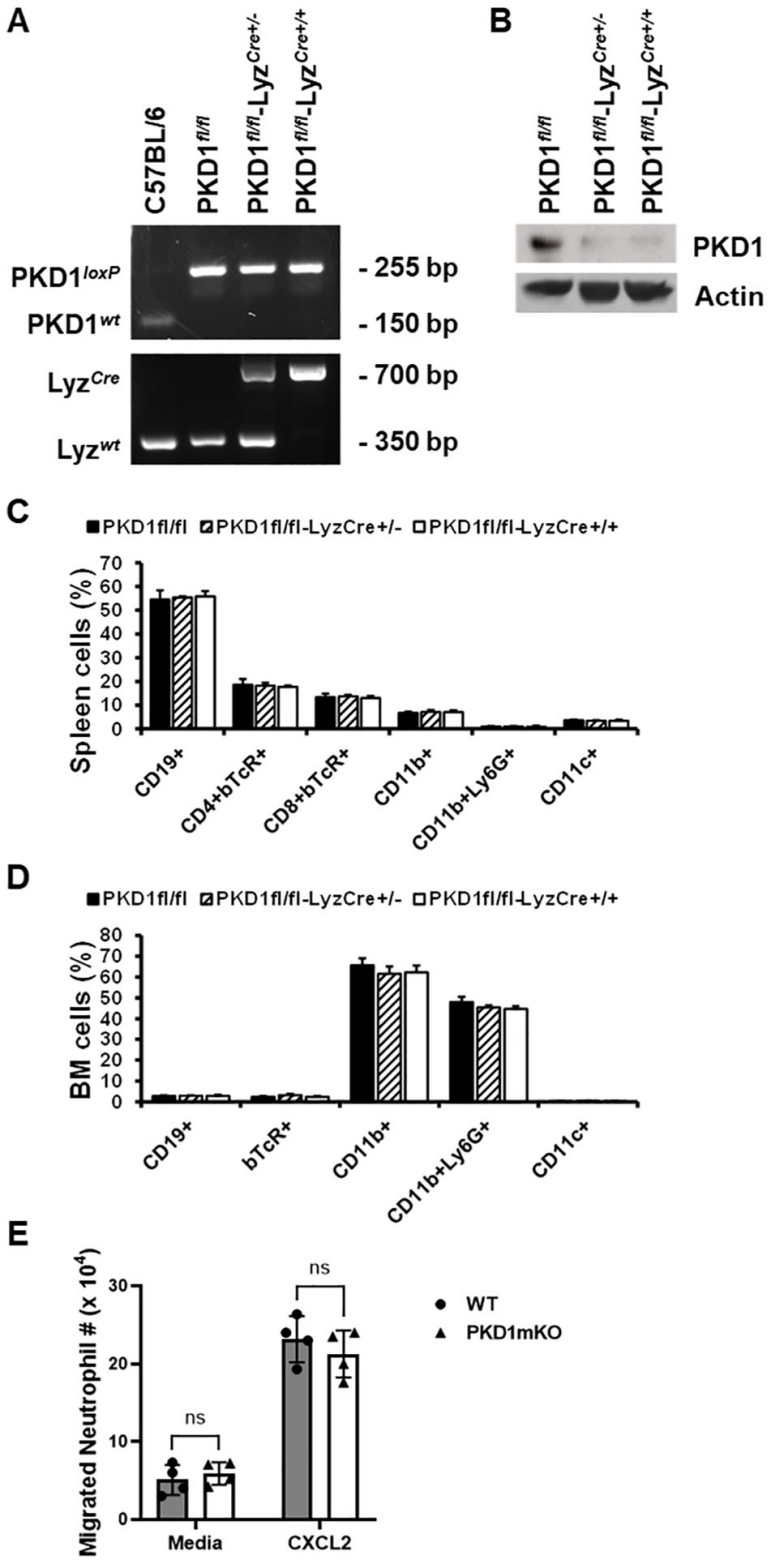
Genotypic and phenotypic analysis of myeloid lineage cell-specific PKD1-deficient mice. **(A)** Genomic DNA was isolated from tail and analyzed for PKD1 alleles and Lyz^Cre^ by PCR. PCR products corresponding to PKD1 WT (151 bp), PKD1*^loxP^* (255 bp), Lyz^WT^ (350 bp), and Lyz^Cre^ (700 bp) are shown. **(B)** Neutrophils from bone marrow were isolated. Neutrophil lysates were prepared, and protein levels of PKD1 and actin were detected by Western blot assay. Actin was used as a loading control. **(C, D)** For immune cell profiling, cells isolated from spleen **(C)** and bone marrow **(D)** of PKD1*^fl/fl^* (wild type; WT; *n* = 5), PKD1*^fl/fl^*-Lyz*^Cre+/-^* (PKD1mKO; *n* = 3), or PKD1*^fl/fl^*-Lyz*^Cre+/+^* (PKD1mKO; *n* = 4) were analyzed by flow cytometry. Gating strategies for flow cytometric analysis of spleen cells and bone marrow (BM) cells are shown in **Supplementary Figures 1A, B**, respectively. Frequency of each cell population (mean % ± SD) is expressed as the percentage of CD45^+^ cells. Statistically significant differences determined by one-way ANOVA with Tukey’s *post-hoc* test. **(E)** One milliliter of media or murine recombinant CXCL2 (50 ng/mL) was placed into the bottom chamber of each designated well in a 24-well plate and 5 × 10^5^ bone marrow neutrophils (isolated from PKD1*^fl/fl^* or PKD1*^fl/fl^*-Lyz*^Cre+/-^*) in 200 µL of media were placed into the top chamber. Two hours later, neutrophils that migrated into the bottom chamber and the bottom chamber side of the membrane were collected and counted. Data represent the mean cell # of quadruplicates ± SD. Significance was determined by two-tailed Student’s *t*-test. ns, not significant.

To delete PKD1 gene in myeloid lineage cells, PKD1*^fl/fl^* mice (mice with inserted lox-p sites in PKD1 gene to flanking exons 12 through 14 that encode the kinase function) (65) were cross-bred with Lyz*^Cre^* mice that express Cre recombinase in myeloid lineage cells, including monocytes, mature macrophages, and granulocytes (64). Resulting myeloid lineage cell PKD1-deficient mice (PKD1*^fl/fl^*-Lyz*^Cre^*) were identified by genotyping (**Figure 1A**). The PKD1*^fl/fl^*-Lyz*^Cre^* mice (both PKD1*^fl/fl^*-Lyz*^Cre+/-^* and PKD1*^fl/fl^*-Lyz*^Cre+/+^* strains) are viable and fertile and do not display any gross physical abnormalities. PKD1 gene deletion and lack of PKD1 mRNA expression in peritoneal macrophages, but not in thymocytes, of PKD1*^fl/fl^*-Lyz*^Cre^* was also detected (**Supplementary Figure 5**). To further confirm PKD1 deletion in myeloid lineage cells, neutrophils were purified from bone marrow cells and then protein levels of PKD1 were analyzed by Western blot assay. As shown in **Figure 1B**, levels of PKD1 protein were substantially reduced in neutrophils isolated from PKD1*^fl/fl^*-Lyz*^Cre^* mice. To evaluate whether PKD1 deletion in myeloid lineage cells affected the general immune cell profile, spleen cells and bone marrow cells were analyzed by flow cytometry. Proportions of CD19^+^ B cells, CD4^+^ T cells, CD8^+^ T cells, CD11b^+^ cells, CD11b^+^Ly6G^+^ cells, and CD11c^+^ cells in the spleens and bone marrow of PKD1mKO (PKD1*^fl/fl^*-Lyz*^Cre+/-^* or PKD1*^fl/fl^*-Lyz*^Cre+/+^*) mice were comparable to those of PKD1*^fl/fl^* mice (**Figures 1C, D**). A previous study has demonstrated that PKD3, one of PKD family members, is required for the neutrophil migration (69). Thus, to evaluate whether deletion of PKD1 affects chemotaxis of neutrophils, the migratory ability of neutrophils was analyzed using trans-well migration assay. As shown in **Figure 1E**, the migratory capability of PKD1-deficient (PKD1^-/-^) neutrophils toward chemokine CXCL2 was comparable to that of PKD1-intact neutrophils, indicating that PKD1^-/-^ neutrophils do not have an intrinsic mobility defect. Taken together, these data demonstrate that PKD1 is deleted in myeloid lineage cells in PKD1*^fl/fl^*-Lyz*^Cre^* mice and that PKD1 deletion in myeloid lineage cells in mice did not significantly alter the general splenic and bone marrow immune cell profiles in mouse and neutrophil’s chemotaxis capacity. These results also indicate that the PKD1*^fl/fl^*-Lyz*^Cre^* mouse can be a suitable tool for investigating the contribution of PKD1 activation in myeloid lineage cells, including neutrophils and macrophages, to the development of acute pulmonary inflammation and HP caused by inhalation of *S. rectivirgula*.

### Effects of protein kinase D1 deletion in myeloid lineage cells on the acute proinflammatory responses in the lung after *S. rectivirgula* inhalation

PKD1 is activated in the lung and plays a pivotal role in acute pulmonary inflammation and HP following inhalation of SR (43, 57). Using cell lines and PKD1-specific siRNA, we found that *S. rectivirgula* activates PKD1 in alveolar type II epithelial cells, neutrophils, and alveolar macrophages, and that PKD1 is indispensable for *S. rectivirgula*-induced expression of cytokines and chemokines in these cells (43). Neutrophils are the predominant cells infiltrated into the lung within several hours following inhalation of *S. rectivirgula* and play an indispensable role in both the acute lung injury and the development of HP (43, 44, 63, 70). To investigate whether lack of PKD1 activation in myeloid lineage cells, such as neutrophils and macrophages, influences inflammatory responses in the lung following exposure to *S. rectivirgula*, PKD1-sufficient mice (WT: PKD1*^fl/fl^*) and myeloid lineage cell PKD1-deficient mice (PKD1mKO: PKD1*^fl/fl^*-Lyz*^Cre^*) were exposed to *S. rectivirgula* by intranasal instillation. Two, six, or twenty-four hours later, BALF and lung tissues were obtained and inflammatory responses to *S. rectivirgula* were assessed by analyzing mRNA levels and protein levels of selected cytokines and chemokines in lungs and BALF, respectively.

**Figure 2:**
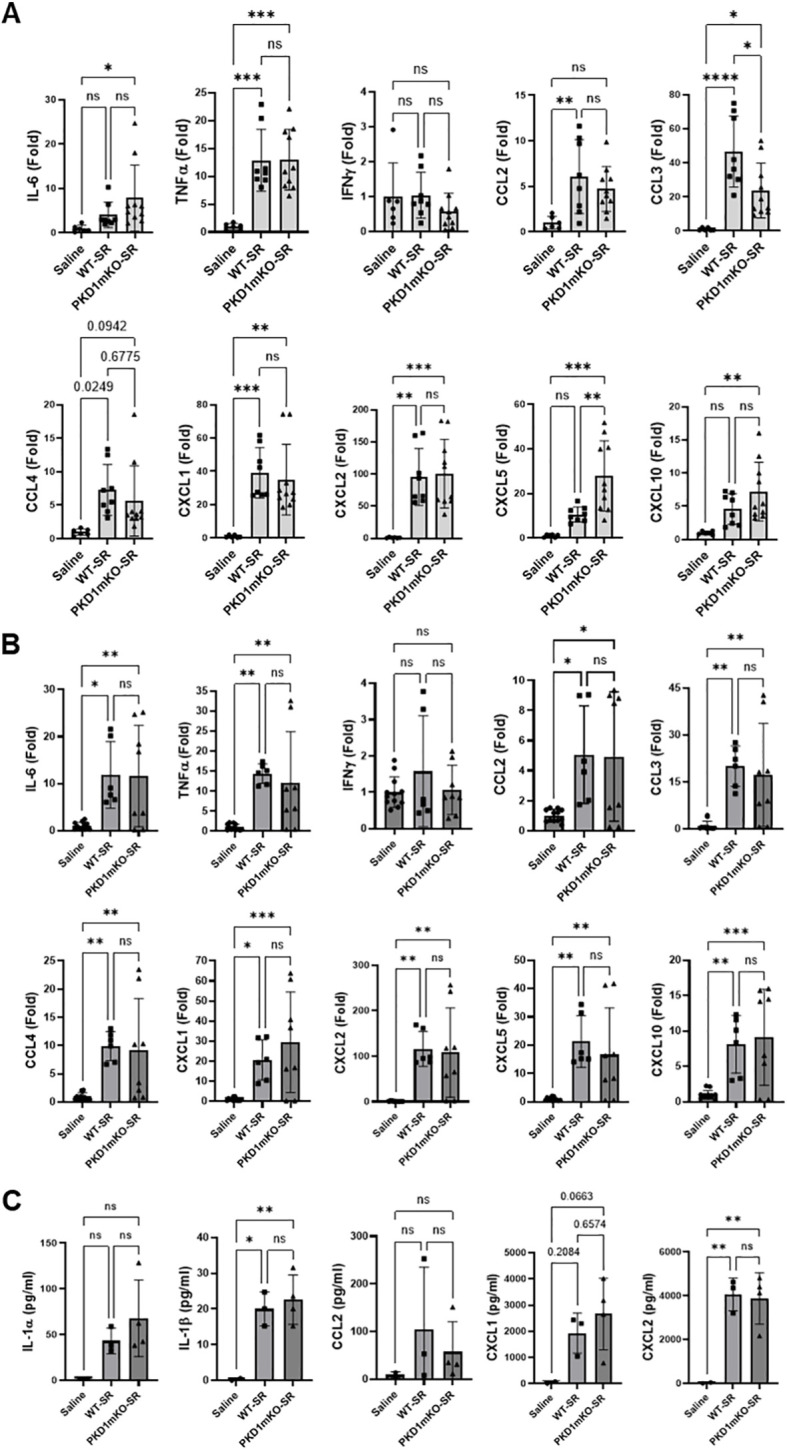
PKD1 in myeloid lineage cells is dispensable for the initial cytokine and chemokine expression in the lungs of mice in response to *S. rectivirgula* inhalation. PKD1*^fl/fl^* mice (WT) and PKD1*^fl/fl^*-LyZ*^Cre^* mice (PKD1mKO) were exposed intranasally to saline or *S. rectivirgula* (100 μg) for 2 h **(A)** or 6 h **(B, C)**. **(A, B)** Total RNA was purified from lung lobes isolated from each individual mouse and reverse transcribed, and then mRNA levels of the indicated genes were analyzed in duplicate by real-time qPCR using SYBR Green Assay. The data on genes that were differentially expressed were normalized to the expression of the housekeeping gene [Actin for panel **(A)** and GAPDH for panel **(B)**]. Fold change comparing *S. rectivirgula*-exposed WT mice and *S. rectivirgula*-exposed PKD1mKO mice to control saline-exposed mice were calculated by comparative quantification algorithm-delta delta Ct method (fold difference = 2^−ΔΔCt^). Data represent the mean (Fold) ± SD. **(C)** Bronchoalveolar lavage (BAL) was performed. Levels of the indicated cytokines and chemokines in BAL fluid were detected by multiplex sandwich assay. Data present the mean concentration (pg/mL) ± SD. Number of mice used for each group is as follows: Saline, *n* = 2 to 6; WT-SR, *n* = 3 to 4; PKD1mKO-SR, *n* = 4 to 5. Statistically significant difference determined by one-way ANOVA with Tukey’s *post-hoc* test is indicated (*p < 0.05; **p < 0.01; ***p < 0.001; ****p < 0.0001). ns, not significant.

At 2 h after the *S. rectivirgula* inhalation, lung mRNA expression levels of TNFα, CCL2, CCL4, CXCL1, and CXCL2 in WT mice and PKD1mKO were significantly increased compared to those in control saline-inhaled mice (**Figure 2A**). Expression levels of those cytokines and chemokines were not significantly different between WT mice and PKD1mKO. These results indicate that PKD1 activation in myeloid lineage cells is dispensable for the initial expression of TNFα, CCL2, CCL4, CXCL1, and CXCL2 in the lungs following *S. rectivirgula* inhalation. These results also suggest that the initial lung cellular sources of these cytokines and chemokines in response to *S. rectivirgula* inhalation may not be the lung-resident myeloid lineage cells. However, we cannot rule out the possibility that other signaling pathways independent of PKD1 may be involved in the expression of those proinflammatory genes in the lung myeloid lineage cells immediately following the *S. rectivirgula* inhalation. Lung mRNA expression levels of CCL3 in both WT mice and PKD1mKO mice 2 h after the *S. rectivirgula* inhalation were also significantly increased compared to those in control saline-inhaled mice. However, lung mRNA expression levels of CCL3 in PKD1mKO mice at 2 h post-*S. rectivirgula* inhalation were significantly lower than those in WT mice, indicating that PKD1 in myeloid lineage cells contributes to the initial expression of CCL3 in the lungs following *S. rectivirgula* inhalation. Lung mRNA expression levels of IL-6, CXCL5, and CXCL10 were slightly but not significantly increased in WT mice compared to those in control mice. However, mRNA expression levels of IL-6, CXCL5, and CXCL10 in PKD1mKO mice at 2 h post-*S. rectivirgula* inhalation were significantly increased compared to those in saline-inhaled control mice. When compared to WT mice, lung mRNA expression levels of IL-6 and CXCL10 in PKD1mKO mice at 2 h post-*S. rectivirgula* inhalation were slightly, but not significantly, increased (*p* = 0.2888 for IL-6; *p* = 0.2147). Lung mRNA expression levels of CXCL5 in PKD1mKO mice at 2 h post-*S. rectivirgula* inhalation were significantly increased compared to CXCL5 levels in WT mice. These indicate that PKD1 activation in myeloid lineage cells is dispensable for the initial expression of IL-6, CXCL5, and CXCL10 in the lungs following *S. rectivirgula* inhalation. These results also suggest a possibility for the presence of PKD1-dependent regulatory mechanism in myeloid lineage cells that negatively affect the initial expression of IL-6, CXCL5, and CXCL10 in the lungs following *S. rectivirgula* inhalation.

At 6 h after the *S. rectivirgula* inhalation, lung mRNA expression levels of IL-6, TNFα, CCL2, CCL3, CCL4, CXCL1, CXCL2, CXCL5, and CXCL10 in both WT mice and PKD1mKO were significantly increased compared to those in control saline-inhaled mice (**Figure 2B**). Expression levels of those cytokines and chemokines in lungs were not significantly different between WT mice and PKD1mKO at 6 h post-*S. rectivirgula* inhalation. IFNγ mRNA expression was not detected at this time point. Protein levels of IL-1β and CXCL2 in BALF were significantly increased in WT and PKD1mKO at 6 h post-exposure to *S. rectivirgula* compared to those in mice exposed to saline (**Figure 2C**). Protein levels of IL-1α, CCL2, and CXCL1 in BALF were also increased in both WT and PKD1mKO exposed to *S. rectivirgula* compared to those in saline-exposed mice but differences were statistically insignificant. Protein levels of these cytokines and chemokines in BALF obtained 6 h after the *S. rectivirgula* inhalation were not statistically different between WT and PKD1mKO. Collectively, these early time point (2 h and 6 h post-challenge) results indicate that PKD1 activation in myeloid lineage cells is dispensable for the initial proinflammatory responses in the lungs following *S. rectivirgula* inhalation.

**Figure 3:**
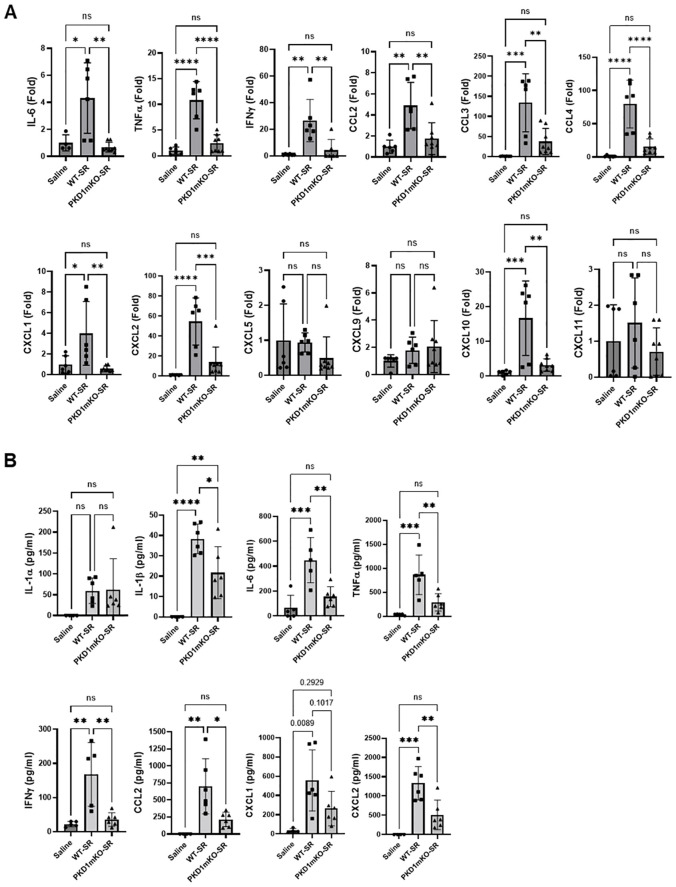
Contribution of PKD1 in myeloid lineage cells to the proinflammatory responses in the lung of mice following one-time exposure to *S. rectivirgula*. PKD1*^fl/fl^* mice (WT) and PKD1*^fl/fl^*-LyZ*^Cre^* mice (PKD1mKO) were exposed intranasally to saline or *S. rectivirgula* (80 μg) for 24 (h) **(A)** Total RNA was purified from lung lobes isolated from each individual mouse and reverse transcribed, and then mRNA levels of the indicated genes were analyzed in duplicate by real-time qPCR using SYBR Green Assay. The data on genes that were differentially expressed were normalized to the expression of the housekeeping gene, GAPDH. Fold change comparing *S. rectivirgula*-exposed WT mice and *S. rectivirgula*-exposed PKD1mKO mice to control saline-exposed mice were calculated by comparative quantification algorithm-delta delta Ct method (Fold difference = 2^−ΔΔCt^). Data represent the mean (Fold) ± SD. **(B)** Bronchoalveolar lavage (BAL) was performed. Levels of the indicated cytokines and chemokines in BAL fluid were detected by either ELISA (IL-6, TNFα, and IFNγ) or multiplex sandwich assay (IL-1α, IL-1β, CCL2, CXCL1, and CXCL2). Data represent the mean concentration (pg/mL) ± SD. Number of mice used for each group is as follows: Saline, *n* = 3 to 5; WT-SR, *n* = 3 to 6; PKD1mKO-SR, *n* = 4 to 6. Statistically significant difference determined by one-way ANOVA with Tukey’s *post-hoc* test is indicated (*p < 0.05; **p < 0.01; ***p < 0.001; ****p < 0.0001). ns, not significant.

At 24 h after the *S. rectivirgula* inhalation, the expression levels of proinflammatory cytokines IL-6, TNFα, and IFNγ and chemokines CCL2, CCL3, CCL4, CXCL1, CXCL2, and CXCL10 in the lungs of WT mice were significantly increased (**Figure 3A**). We were not able to detect increased expression of CXCL5, CXCL9, and CXCL11 in the lungs of WT mice at 24 h after the *S. rectivirgula* exposure. When compared to WT, PKD1mKO mice showed significantly reduced mRNA expression levels of TNFα, IL-6, IFNγ, CCL2, CCL3, CCL4, CXCL1, CXCL2, and CXCL10 in the lungs in response to *S. rectivirgula* exposure. The mRNA expression levels of those cytokines and chemokines in the lungs of PKD1mKO exposed to *S. rectivirgula* for 24 h were not significantly different from those in lungs of control mice exposed to saline. Protein levels of IL-1α, IL-1β, IL-6, TNFα, IFNγ, CCL2, CXCL1, and CXCL2 in BALF were significantly increased in WT mice at 24 h after the exposure to *S. rectivirgula* compared to those in mice exposed to saline (**Figure 3B**). Compared to those in WT mice, protein levels of IL-1β, IL-6, TNFα, IFNγ, CCL2, and CXCL2 in BALF from *S. rectivirgula*-exposed PKD1mKO mice were significantly decreased. Although it showed substantial reduction in *S. rectivirgula*-exposed PKD1mKO, the protein levels of CXCL1 in BALF were not significantly different between WT and PKD1mKO exposed to *S. rectivirgula* one time for 24 h (*p* = 0.1017). When compared to those in saline-treated control mice, the protein levels of IL-6, TNFα, IFNγ, CCL2, and CXCL1 in BALF from SR-exposed PKD1mKO were not significantly different. However, the protein levels of IL-1β in PKD1mKO exposed to *S. rectivirgula* were significantly higher than those in saline-treated control mice. Increasing trends for the protein levels of IL-1α (*p* = 0.0878) and CXCL2 (*p* = 0.1091) in PKD1mKO following *S. rectivirgula* exposure, compared to those in saline-treated control, were also observed. These results indicate that PKD1 in myeloid lineage cells is involved in cytokine and chemokine production in the lungs during the later phase of acute lung insult by *S. rectivirgula* exposure. Taken together, our results demonstrate that although multiple signaling pathways and multiple types of lung cells are involved in acute pulmonary inflammation caused by exposure to *S. rectivirgula*, PKD1 in myeloid lineage cells plays a significant role for the acute pulmonary proinflammatory responses following one-time exposure to *S. rectivirgula*.

### Development of acute neutrophilia following single-time exposure to *S. rectivirgula* is significantly suppressed in myeloid lineage cell protein kinase D1-deficient mice

**Figure 4:**
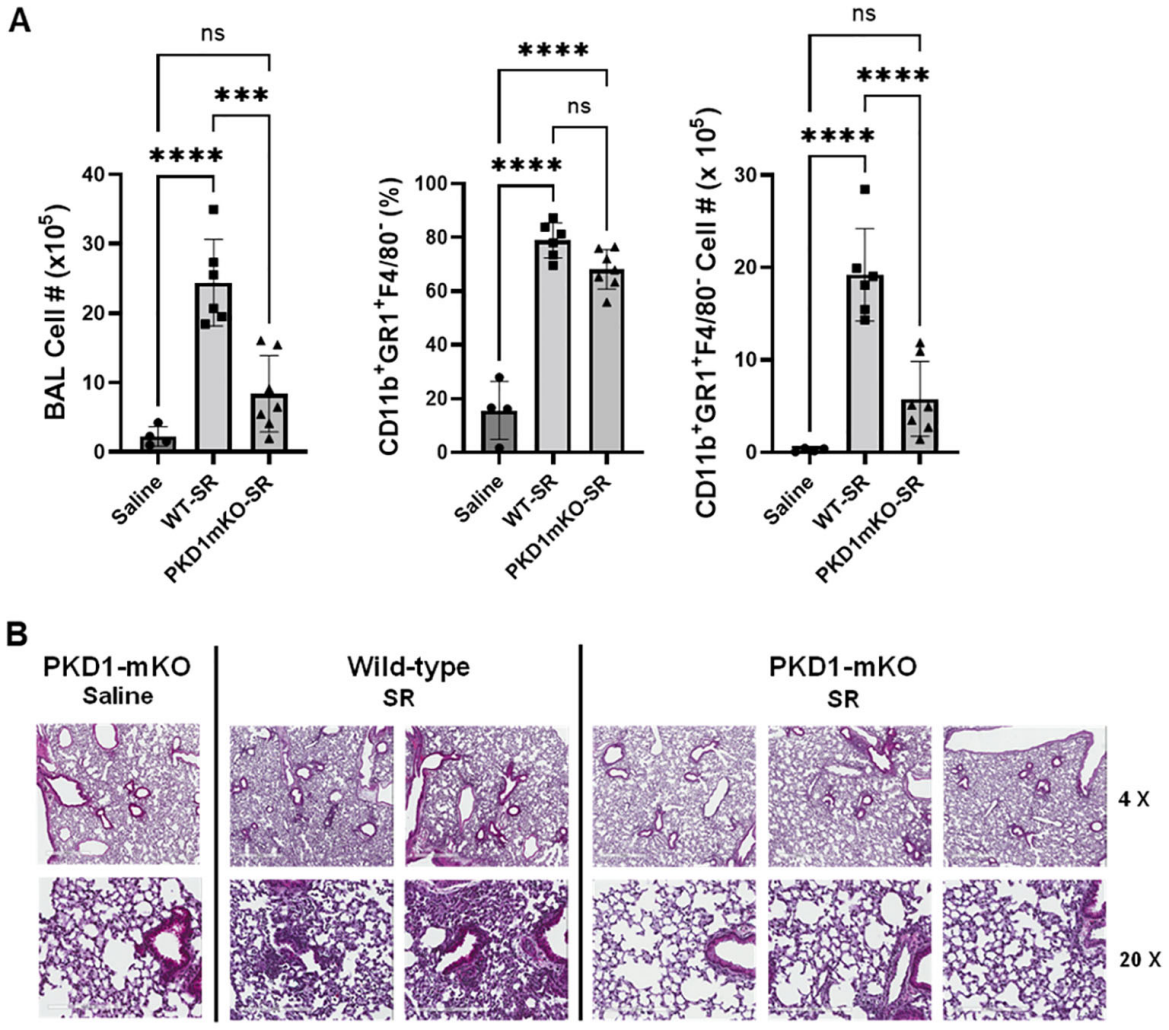
PKD1 in myeloid lineage cells contributes significantly to the neutrophilic alveolitis developed following single exposure to *S. rectivirgula*. PKD1*^fl/fl^* mice (WT) and PKD1*^fl/fl^*-LyZ*^Cre^* mice (PKD1mKO) were exposed intranasally to saline or *S. rectivirgula* (80 μg) for 24 h. **(A)** BAL cells were counted using trypan blue exclusion and presented as the mean cell number ± SD (left panels). Total neutrophil cell count was derived by staining BAL cells with Abs to Gr-1, CD11b, and F4/80 followed by flow cytometric analysis. Gating strategy for flow cytometric analysis is shown in **Supplementary Figure 2**. Neutrophils were identified as CD11b^+^Gr1^+^/F4/80^-^. The frequency of neutrophils is expressed as % in BAL cells, and data represent the mean (%) ± SD (middle panels). The number of neutrophils is presented as the mean cell number ± SD (right panels). Statistical differences were determined by one-way ANOVA with Tukey’s *post-hoc* test and significant differences are indicated (***p < 0.001; ****p < 0.0001). ns, not significant. **(B)** Representative H&E staining of the left lung lobe sections from mice exposed to saline or SR for 24 h are shown. The Aperio ScanScope^®^XT Slide Scanner system was used to capture whole-slide digital images. Each column represents the lung collected from an individual mouse. The images presented are 4× magnification (scale bar = 600 μm) and 20× magnification (scale bar = 200 µm). Number of mice used for each group is as follows: Saline, *n* = 3 to 11; WT-SR, *n* = 3 to 6; PKD1mKO-SR, *n* = 4 to 7.

Significantly reduced expression of CCL2, CCL3, CCL4, CXCL1, and CXCL2 in the lungs of PKD1mKO 24 h after the single exposure to *S. rectivirgula* predict that PKD1 in myeloid lineage cells may contribute to the leukocyte influx into the lung by upregulating these chemokines following exposure to *S. rectivirgula*. Therefore, we investigated whether deletion of PKD1 in myeloid lineage cells affects leukocyte infiltration into the lung following *S. rectivirgula* exposure. As shown in **Figure 4A** (left panel), at 24 h post-exposure to *S. rectivirgula*, WT mice exhibited significantly increased total BAL cell numbers (alveolitis) compared to those in control mice exposed to saline. Histological sections of lungs from *S. rectivirgula*-exposed WT mice also showed the presence of extensive leukocyte infiltration in the lungs compared to those from saline-exposed mice (**Figure 4B**). As previously reported (43, 44, 57, 63, 70), neutrophils were the predominant cell type recovered from the airways in WT mice exposed to *S. rectivirgula* (**Figure 4A**, middle panel). Approximately 78% of cells recovered from BALF were neutrophils determined by flow cytometric analysis. In contrast, significantly less BAL cells were recovered from the PKD1mKO mice at 24 h after the *S. rectivirgula* exposure compared to the *S. rectivirgula*-exposed WT mice (**Figure 4A**, left panel). The number of total cells recovered from the BALF from *S. rectivirgula*-exposed PKD1mKO mice were approximately 35% of those in *S. rectivirgula*-exposed WT mice. Histological sections of lungs from *S. rectivirgula*-exposed PKD1mKO mice also showed substantially less leukocyte infiltration in the lungs compared to those from *S. rectivirgula*-exposed WT mice (**Figure 4B**). Like the *S. rectivirgula*-exposed WT mice, the major cell type recovered from the airways of PKD1mKO mice was neutrophils (approximately 67%) (**Figure 4A**, middle panel). Although frequencies of neutrophils in BAL cells from *S. rectivirgula*-exposed PKD1-mKO mice were slightly lower compared to those of *S. rectivirgula*-exposed WT mice, the difference was not significant (*p* = 0.0704). The number of neutrophils recovered from the BALF from PKD1mKO mice were approximately 31% of those in *S. rectivirgula*-exposed WT mice (**Figure 4A**, right panel). Although they are not statistically significant, the numbers of total cells (*p* = 0.1849) and neutrophils (*p* = 0.1055) recovered from BALF of *S. rectivirgula*-exposed PKD1mKO were substantially higher than those in saline-exposed mice. Taken together, our results demonstrate that PKD1 in myeloid lineage cells plays a significant role in *S. rectivirgula*-induced acute neutrophilic alveolitis.

### Protein kinase D1 in myeloid lineage cell contributes to leukocyte infiltration into the bronchial space and granuloma formation following repeated exposures to *S. rectivirgula*

**Figure 5:**
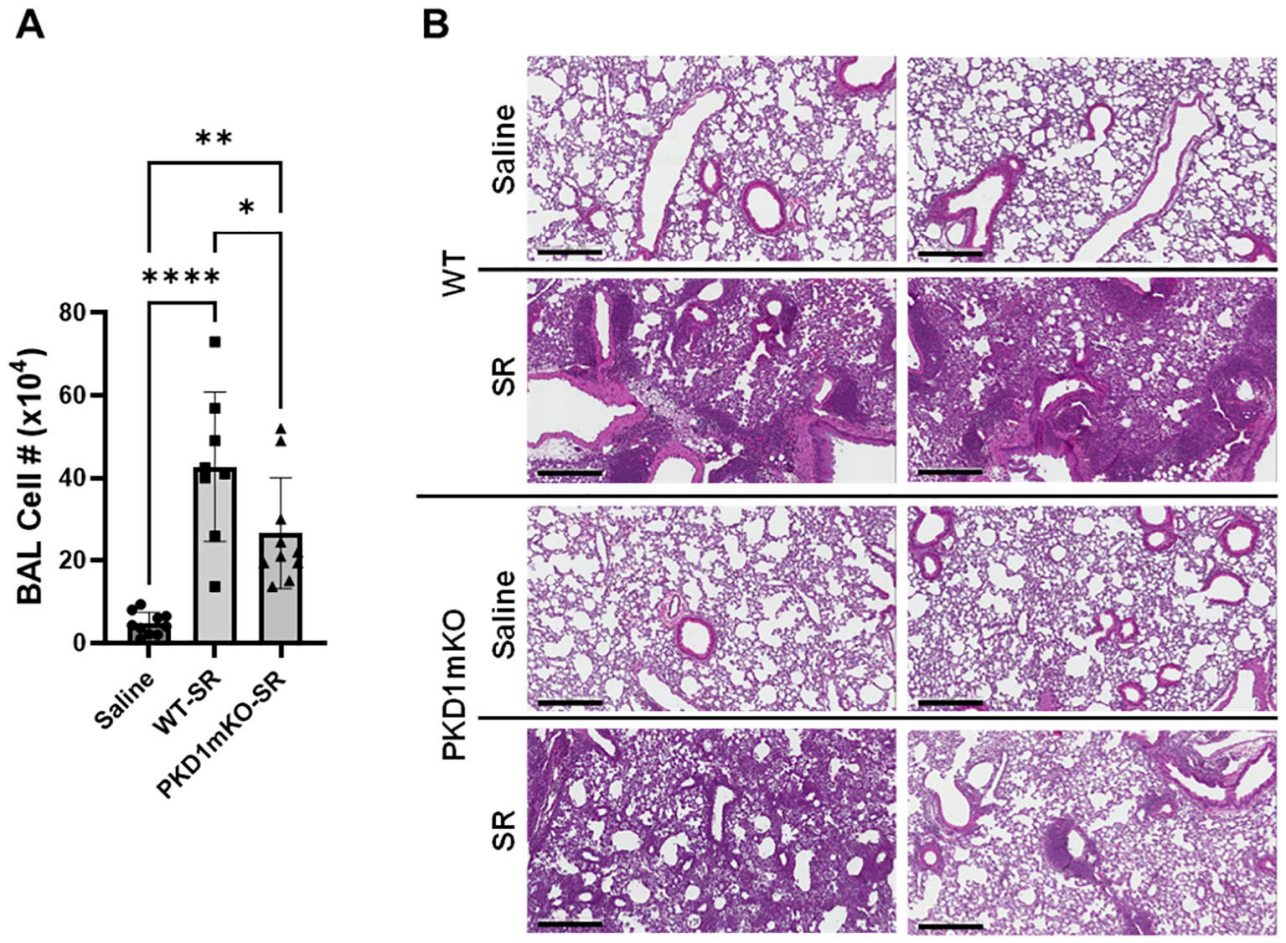
Deletion of PKD1 in myeloid lineage cells results in significant reduction in alveolitis and granuloma formation following repeated exposures to *S. rectivirgula*. PKD1*^fl/fl^* mice (WT) and PKD1*^fl/fl^*-LyZ*^Cre^* mice (PKD1mKO) were exposed intranasally to saline or *S. rectivirgula* (100 μg) three times per week for 3 weeks. **(A)** Seventy-two hours after the last SR exposure, BAL was performed, and the BAL cells recovered. BAL cells were counted to determine the degree of alveolitis using trypan blue exclusion. Data represent the mean cell number ± SD (*n* = 8 to 10 mice/group). Significance was determined by one-way ANOVA with Tukey’s *post-hoc* test. Statistically significant differences are indicated (*p < 0.05; **p < 0.01; ****p < 0.0001). **(B)** Forty-eight hours after the last *S. rectivirgula* exposure, the left lung lobes were removed from the mice. Representative H&E staining of the left lung lobe sections are shown. The Aperio ScanScope^®^XT Slide Scanner system was used to capture whole-slide digital images. Each panel represents the lung collected from an individual mouse. Number of mice used for each group is as follows: Saline, *n* = 4; WT-SR, *n* = 4; PKD1mKO-SR, *n* = 3. The scale bar = 300 µm.

Repeated exposures to HP-inciting antigens cause recurring leukocyte infiltrations into the airways and interstitial lung space and lead to the formation of granulomas in both HP patients and animal models of HP (2, 3, 70, 71). To determine whether PKD1 in myeloid lineage cells contributes to the immune cell influx into the bronchial space and lungs following repeated exposures to *S. rectivirgula*, WT mice and PKD1mKO mice were exposed to *S. rectivirgula* three times (once a day)/week for 3 weeks as previously described (57). As shown in **Figure 5A**, leukocyte infiltration in bronchial space was significantly increased in both WT and PKD1mKO compared to the control saline-exposed mice. However, *S. rectivirgula*-induced leukocyte infiltration in bronchial space was significantly suppressed in PKD1mKO compared to WT (approximately 62% of WT-SR). Cellular composition of alveolitis following 3-week exposures to *S. rectivirgula* was not significantly different between WT mice and PKD1mKO mice (**Table 1**). To determine whether PKD1 in myeloid lineage cells contributes to leukocyte infiltration and accumulation, and the development of granulomas in the lungs, we examined lung tissue sections from WT mice and PKD1mKO mice that had been repeatedly exposed to *S. rectivirgula* for 3 weeks. As shown in **Figure 5B** and **Supplementary Figure 6**, while mice exposed to saline showed normal lung architecture, both WT mice and PKD1mKO mice repeatedly exposed to *S. rectivirgula* for 3 weeks exhibited alveolitis and granuloma formation. However, compared to WT mice, deletion of PKD1 in myeloid lineage cells resulted in substantially less leukocyte accumulation and reduced granuloma formation following repeated exposures to *S. rectivirgula* (**Figure 5B**; **Supplementary Figure 6**). Of note, the frequencies of CD45^+^ cells in isolated LICs were significantly lower in PKD1mKO mice compared to WT mice (**Supplementary Table 2**). Cellular composition of LICs following 3-week exposures to *S. rectivirgula* was not significantly different between WT mice and PKD1mKO mice (**Supplementary Table 2**). Collectively, these results demonstrate that PKD1 in myeloid lineage cells contributes significantly to leukocyte infiltration and accumulation in the lungs, and subsequent granuloma formation following repeated exposures to *S. rectivirgula*.

**Table 1:** Cellular composition of alveolitis following 3-week exposures of WT and PKD1mKO mice to *S. rectivirgula*.

	AM (%)	MAC (%)	Neutrophils (%)	CD11c+CD11b-F4/80-(%)	CD11c+CD11b+F4/80-(%)	CD4^+^ T (%)	CD8^+^ T (%)
**WT**	40.63 ± 14.64	3.28 ± 1.03	15.05 ± 7.01	2.20 ± 0.73	1.59 ± 0.27	15.78 ± 5.28	6.14 ± 3.33
**PKD1mKO**	41.77 ± 5.30	3.87 ± 1.36	15.39 ± 2.91	2.07 ± 0.40	1.80 ± 0.52	13.07 ± 3.52	3.88 ± 1.07

WT (n = 4) and PKD1mKO (n = 5) were exposed to *S. rectivirgula* (100 μg) three times per week for 3 weeks and analyzed 72 hours after the last exposure. The frequency of alveolar macrophages (AM: CD11c^+^F4/80^+^), macrophages (MAC: CD11b^+^F4/80^+^CD11c^-^), neutrophils (CD11b^+^Gr1^+^F4/80^-^), CD11c^+^CD11b^-^F4/80^-^ cells, CD11c^+^CD11b^+^F4/80^-^ cells, CD4^+^ T cells (CD4^+^βTcR^+^), and CD8^+^ T cells (CD8^+^βTcR^+^) in BAL cells were measured by flow cytometry followed by analysis using FlowJo software as described in Materials and Methods and expressed as % of CD45^+^ cells. Values are given as mean ± SD of each group. Gating strategy is shown in **Supplementary Figure 3A**. Significance was determined by two-tailed Student’s t-test.

### Protein kinase D1 in myeloid lineage cells contributes to the expression of major histocompatibility complex class II in neutrophils and macrophages infiltrated into the bronchial space and lung interstitium following repeated exposures to *S. rectivirgula*

**Figure 6:**
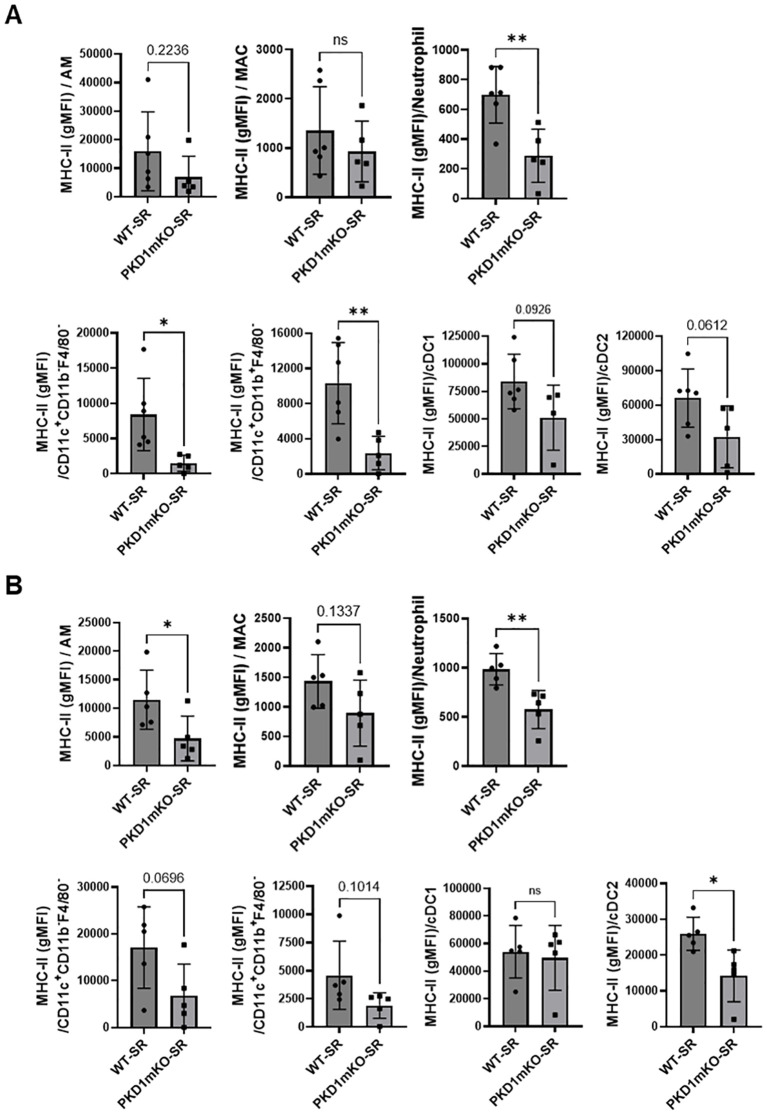
PKD1 in myeloid lineage cells contributes to the expression of MHC-II in neutrophils and macrophages infiltrated into the bronchial space and lung interstitium following repeated exposures to *S. rectivirgula.* WT (*n* = 5 to 6/group) or PKD1mKO mice (*n* = 5/group) were exposed intranasally to *S. rectivirgula* (100 μg) three times per week for 3 weeks. BAL cells **(A)** and LICs **(B)** were isolated from mice at 48 h after the last *S. rectivirgula* exposure. Cells were stained with fluorochrome-conjugated Abs and then analyzed by flow cytometry and FlowJo flow software. Gating strategies for flow cytometric analysis of BAL cells and LICs are shown in **Supplementary Figures 3A, B**, respectively. Levels of surface expression of MHC-II on cells were determined by geometric mean fluorescent intensity (gMFI) of each marker in the indicated cell population. Each dot or square represents individual mouse. Data represent the mean gMFI ± SD. Significance was determined by two-tailed Student’s *t*-test. Statistically significant differences are indicated (*p < 0.05; **p < 0.01). ns, not significant.

In the previous studies, we found that PKD1 contributes significantly to the increased surface expression of major histocompatibility complex class II (MHC-II) on polymorphonuclear cells (PMNs) and alveolar macrophages (AMs) isolated from bronchoalveaolar spaces and lung interstitium of mice repeatedly exposed to *S. rectivirgula* (57). To investigate whether deletion of PKD1 in myeloid lineage cells affects the surface expression of MHC-II on cells in bronchoalveaolar spaces and lung interstitium of the mice repeatedly exposed to *S. rectivirgula*, BAL cells and LICs isolated from WT and PKD1mKO mice repeatedly exposed to *S. rectivirgula* for 3 weeks were analyzed by flow cytometry and the levels of surface expression of MHC-II were measured based on the gMFI in each cell population. As shown in **Figure 6A**, the levels of the surface expression of MHC-II on neutrophils, CD11c^+^CD11b^-^F4/80^-^ cells (contains MHC-II^low^ population and cDC1 population), and CD11c^+^CD11b^+^F4/80^-^ cells (contains MHC-II^low^ population and cDC2 population) isolated from the bronchoalveaolar spaces of PKD1mKO mice were significantly lower than those from WT mice. MHC-II surface expression on AMs, cDC1s, and cDC2s isolated from the bronchoalveaolar spaces of PKD1mKO was substantially, but not significantly (*p* = 0.2236 for AMs, *p* = 0.0926 for cDC1s, and *p* = 0.0612 for cDC2s), lower than that in WT mice. The levels of the surface expression of MHC-II on MACs in the bronchoalveaolar spaces were comparable between WT and PKD1mKO. The levels of the surface expression of CD86 on AMs and MACs were slightly lower in PKD1mKO mice compared to those in WT mice (*p* = 0.0616 for AMs and *p* = 0.0981 for MACs) (**Supplementary Figure 7**). In contrast, the levels of the surface expression of CD86 on neutrophils isolated from the bronchoalveaolar spaces of PKD1mKO mice were slightly, but significantly, higher than those from WT mice (**Supplementary Figure 7**). The surface expression levels of CD86 on CD11c^+^CD11b^-^F4/80^-^ cells and CD11c^+^CD11b^+^F4/80^-^ cells, and the surface expression levels of CD40 and CD206 on AMs, MACs, neutrophils, CD11c^+^CD11b^-^F4/80^-^ cells, and CD11c^+^CD11b^+^F4/80^-^ cells isolated from the bronchoalveaolar spaces were comparable between WT mice and PKD1mKO mice exposed to *S. rectivirgula* for 3 weeks (**Supplementary Figure 7**).

Surface expression levels of MHC-II on AMs, neutrophils, and cDC2s isolated from lung interstitium of PKD1mKO mice repeatedly exposed to *S. rectivirgula* were significantly lower than those from WT mice (**Figure 6B**). MHC-II surface expression on MACs, CD11c^+^CD11b^-^F4/80^-^ cells, and CD11c^+^CD11b^+^F4/80^-^ cells isolated from lung interstitium of PKD1mKO was substantially, but not significantly (*p* = 0.1337 for MACs, *p* = 0.0696 for CD11c^+^CD11b^-^F4/80^-^ cells, and *p* = 0.1014 for CD11c^+^CD11b^+^F4/80^-^ cells), lower than that in WT mice. The surface expression levels of MHC-II on cCD1s, and CD86, CD40, and CD206 on AMs, MACs, neutrophils, CD11c^+^CD11b^-^F4/80^-^ cells, and CD11c^+^CD11b^+^F4/80^-^ cells isolated from lung interstitium of PKD1mKO mice repeatedly exposed to *S. rectivirgula* were comparable to those of WT mice (**Supplementary Figure 8**). Taken together, our results indicate that PKD1 in myeloid lineage cells may affect the level of the surface expression of MHC-II in myeloid lineage cells in the lungs during the development of HP caused by repeated exposures to *S. rectivirgula*.

### Protein kinase D1 in myeloid lineage cells contributes to the expression of T helper 1- and T helper 17-related proinflammatory cytokines and chemokines in the lungs following repeated exposures to *S. rectivirgula*

**Figure 7:**
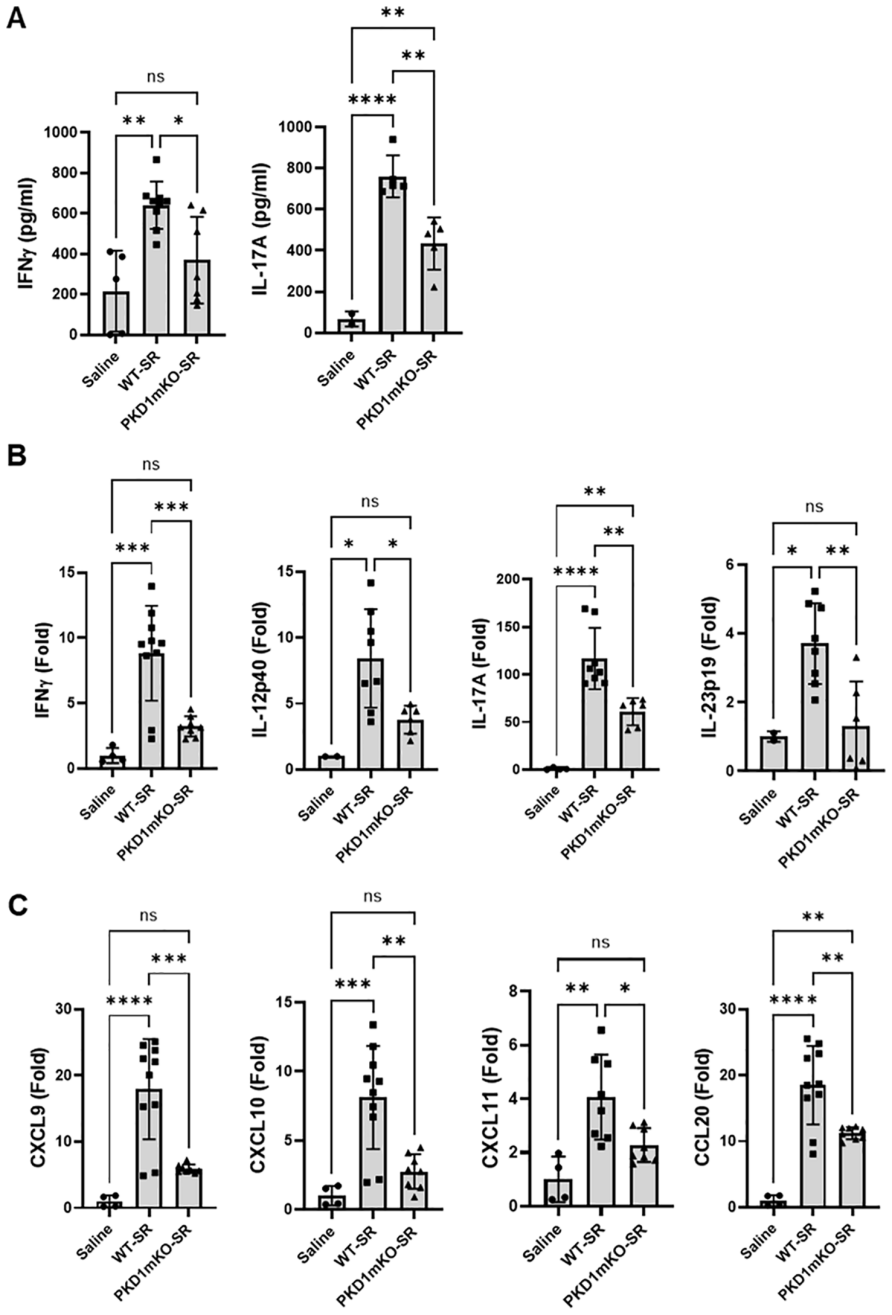
PKD1 in myeloid lineage cells contributes to the increased expression of Th1/Th17-related cytokines and chemokines in lungs following repeated exposures to *S. rectivirgula*. PKD1*^fl/fl^* mice (WT) and PKD1*^fl/fl^*-LyZ*^Cre^* mice (PKD1mKO) were exposed intranasally to saline or *S. rectivirgula* (100 μg) three times per week for 3 weeks. Forty-eight hours after the last *S. rectivirgula* exposure, BALF and the lungs were collected. **(A)** Levels of the indicated cytokines IFNγ and IL-17A in BALF were detected by ELISA. Data represent the mean (pg/mL) ± SD. Each symbol represents individual mouse. Significance was determined by one-way ANOVA with Tukey’s *post-hoc* test. Statistically significant differences are indicated (*p < 0.05; **p < 0.01; ****p < 0.0001). ns = not significant. Number of mice used for each group is as follows: Saline, *n* = 2 to 5; WT-SR, *n* = 5 to 9; PKD1mKO-SR, *n* = 5 to 7. **(B, C)** Total RNA was purified from lung lobes isolated from each individual mouse and reverse transcribed. mRNA levels of the indicated genes were analyzed in duplicates by RT-qPCR using SYBR Green Assay. The data on genes that were differentially expressed was normalized to the expression of the housekeeping gene, GAPDH. Fold change comparing SR-treated exposed WT mice and SR-treated PKD1mKO mice to control mice exposed to saline was calculated by comparative quantification algorithm-delta delta Ct method (Fold difference = 2^−ΔΔCt^). Data represent the mean (Fold) ± SD. Significance was determined by one-way ANOVA with Tukey’s *post-hoc* test. Statistically significant differences are indicated (*p < 0.05; **p < 0.01; ***p < 0.001; ****p < 0.0001). ns, not significant. Number of mice used for each group is as follows: Saline, *n* = 1 to 2; WT-SR, *n* = 4 to 5; PKD1mKO-SR, *n* = 3 to 4.

Previous studies have shown that Th1- and Th17-associated cytokines and chemokines are critical to the development and severity of HP (33, 72, 73). Using inducible systemic PKD1-knockout mice, we previously found that PKD1 contributes substantially to the expression of IL-17A and Th1/Th17-associated chemokine CXCL9 following repeated exposures to *S. rectivirgula* (57). A pilot screening of 40 cytokines/chemokines in BALFs obtained from WT mouse and PKD1mKO mouse exposed repeatedly to *S. rectivirgula* for 3 weeks using a membrane-based mouse cytokine array showed that levels of triggering receptor expressed on myeloid cells-1 (TREM-1), IFNγ, IL-1α, IL-1β, IL-16, and IL-17A are lower in the *S. rectivirgula*-exposed PKD1mKO mouse compared to those in the *S. rectivirgula*-exposed WT mouse (**Supplementary Figure 9**). In contrast, levels of tissue inhibitor of metalloproteinase-1 (TIMP-1), macrophage colony stimulating factor (M-CSF), CCL1, and IL-7 are higher in the *S. rectivirgula*-exposed PKD1mKO mouse than in the *S. rectivirgula*-exposed WT mouse. This result indicates a possibility that PKD1 in myeloid lineage cells may contribute to promoting the inflammatory Th1 and Th17 environment in the lung in HP. To further investigate whether deletion of PKD1 in myeloid lineage cells affects the expression of Th1- and Th17-related cytokines and chemokines in the lungs following repeated exposures to *S. rectivirgula*, we analyzed protein and mRNA expression levels of Th1- and Th17-related cytokines and chemokines in the BALF and lung tissues of WT mice and PKD1mKO mice repeatedly exposed to *S. rectivirgula* for 3 weeks. As shown in **Figures 7A, B**, the levels of both protein (in BALF) and mRNA (in lung tissue) of IFNγ and IL-17A in WT mice following repeated exposures to *S. rectivirgula* were significantly increased compared to those in saline-exposed control mice. In contrast, the levels of IFNγ in BALF and lungs of PKD1mKO mice exposed to *S. rectivirgula* repeatedly for 3 weeks were not significantly different from those from saline-exposed control mice. The levels of IL-17A (both protein and mRNA) were significantly higher in *S. rectivirgula*-exposed PKD1mKO than in saline-exposed control. When compared to those in *S. rectivirgula*-exposed WT mice, the levels of IFNγ and IL-17A in BALF and lung tissue obtained from PKD1mKO mice repeatedly exposed to *S. rectivirgula* were significantly reduced (**Figures 7A, B**). The mRNA levels of Th1/Th17-related cytokine IL-12p40 and IL-23p19 in the lung tissues of PKD1mKO mice repeatedly exposed to *S. rectivirgula* for 3 weeks were significantly reduced compared to those in *S. rectivirgula*-exposed WT mice, but comparable to those in saline-exposed control (**Figure 7B**). The mRNA levels of proinflammatory cytokine TNFα in lungs of PKD1mKO mice repeatedly exposed to *S. rectivirgula* for 3 weeks were significantly reduced compared to those in *S. rectivirgula*-exposed WT mice, but significantly higher than those in saline-exposed control (**Supplementary Figure 10A**). IL-6 mRNA levels in the lungs were not significantly different between saline-exposed control mice, *S. rectivirgula*-exposed WT mice, and *S. rectivirgula*-exposed PKD1mKO under our experimental condition (**Supplementary Figure 10A**). Taken together, these results indicate that although it is not indispensable, PKD1 in myeloid lineage cells plays a significant role in the expression of Th1- and Th17-related cytokines in mouse lungs following repeated exposures to *S. rectivirgula*.

Chemokines regulate leukocyte migration, differentiation, and activation by interacting with specific receptors expressed on surface of leukocytes. Chemokines CXCL9, CXCL10, and CXCL11 interact with cell surface chemokine receptor CXCR3 and contribute to Th1 polarization (74). Chemokine CCL20 binds to chemokine receptor CCR6 and contribute to the Th17 polarization (75). We further investigate whether PKD1 in myeloid linage cells contributes to the *S. rectivirgula*-mediated expression of these chemokines involved in Th1/Th17 polarization. As demonstrated in **Figure 7C**, levels of mRNA expression of CXCL9, CXCL10, CXCL11, and CCL20 in the lungs of WT mice repeatedly exposed to *S. rectivirgula* for 3 weeks were significantly increased compared to those of control mice exposed to saline for 3 weeks. Compared to the expression level in WT mice, expression levels of CXCL9, CXCL10, and CXCL11 in lungs of PKD1mKO mice following repeated exposure to *S. rectivirgula* were significantly suppressed. Expression levels of CXCL9, CXCL10, and CXCL11 in lungs of PKD1mKO mice exposed to *S. rectivirgula* repeatedly for 3 weeks were not significantly different from those from saline-exposed control mice. The mRNA levels of CCL20 in lungs of PKD1mKO mice repeatedly exposed to *S. rectivirgula* for 3 weeks were significantly reduced compared to those in *S. rectivirgula*-exposed WT mice, but significantly higher than those in saline-exposed control. The mRNA expression levels of CCL5, CXCL12, and CXCL16 in the lungs were not different between saline-exposed control mice, *S. rectivirgula*-exposed WT mice, and *S. rectivirgula*-exposed PKD1mKO mice under our experimental condition (**Supplementary Figure 10B**). Taken together, these results demonstrate that PKD1 in myeloid lineage cells contributes significantly to the expression of Th1- and Th17-related cytokines IFNγ, IL-12, IL-17A, and IL-23 and Th1- and Th17-polarizing chemokines CXCL9, CXCL10, CXCL11, and CCL20 following repeated exposures to *S. rectivirgula*. Our results indicate that PKD1 in myeloid lineage cells might play a pivotal role in the influx and development of pathogenic Th1 and Th17 cells in the lungs in HP caused by repeated exposures to *S. rectivirgula*.

### Protein kinase D1 in myeloid lineage cells contributes to the nonconventional T helper 1 cell accumulation in the lungs following repeated exposures to *S. rectivirgula*

**Figure 8:**
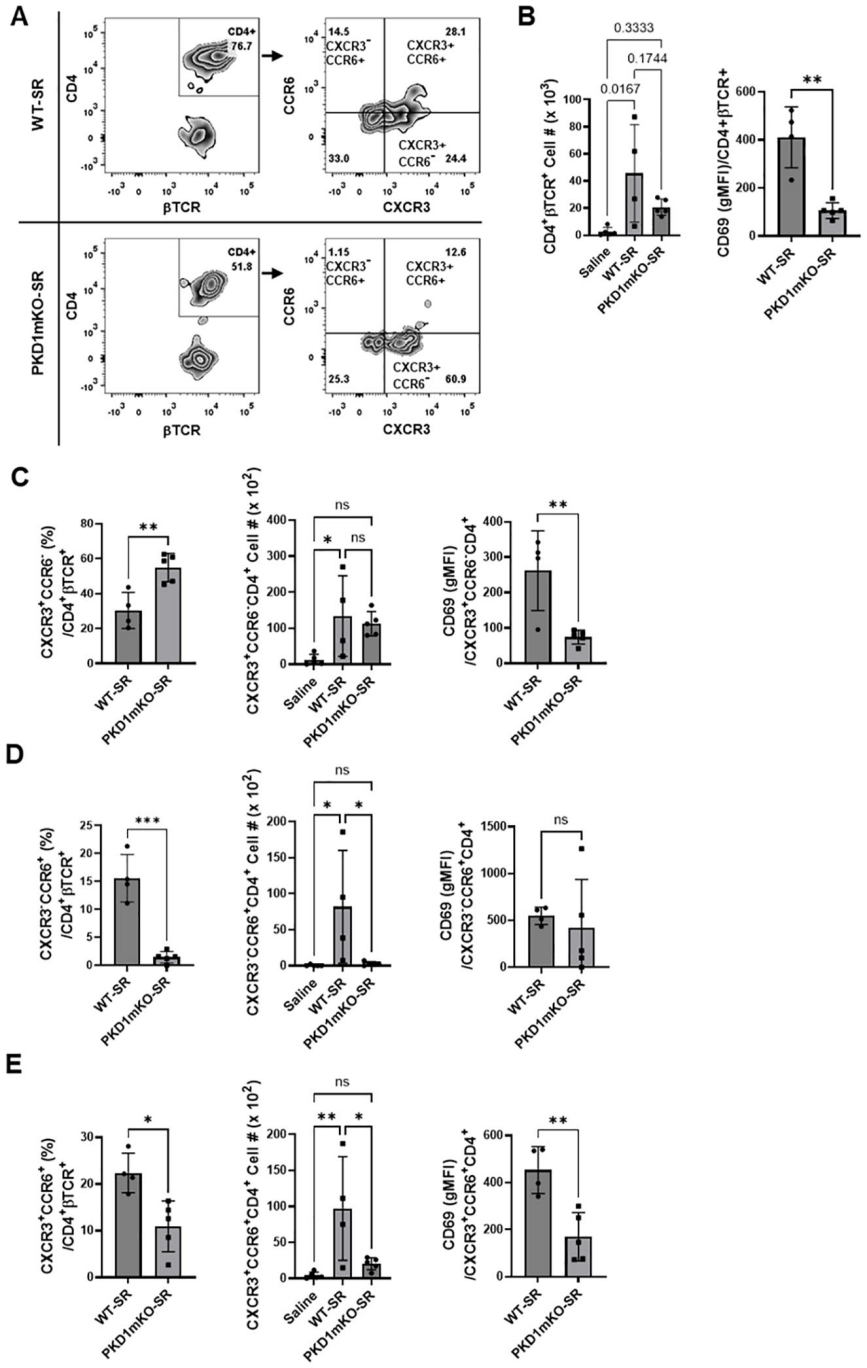
Effects of myeloid lineage cell-specific PKD1 on CXCR3^+^CCR6^+^ cell accumulation in the lungs following repeated exposures to *S. rectivirgula*. PKD1*^fl/fl^* mice (WT) and PKD1*^fl/fl^*-LyZ*^Cre^* mice (PKD1mKO) were exposed intranasally to saline or SR (100 μg) three times per week for 3 weeks. Seventy-two hours after the last *S. rectivirgula* challenge, BAL cells were stained with fluorochrome-conjugated Abs and then analyzed by flow cytometry and FlowJo flow software. Gating strategies for flow cytometric analysis of CD4^+^ T cells in BAL cells are shown in **Supplementary Figure 3A**. **(A)** Gating of CD4^+^ T cells for CXCR3 and CCR6 expression. **(B–E)** The frequency of CXCR3^+^CCR6^-^ cells, CXCR3^-^CCR6^+^ cells, or CXCR3^+^CCR6^+^ cells in the CD4^+^ T-cell population is expressed as % in the CD4^+^βTCR^+^ cell population. The number of CD4^+^βTCR^+^ cells, CXCR3^+^CCR6^-^CD4^+^βTCR^+^ cells, CXCR3^-^CCR6^+^CD4^+^βTCR^+^ cells, and CXCR3^+^CCR6^+^CD4^+^βTCR^+^ recovered from BAL was derived by staining BAL cells with Abs to CXCR3, CCR6, CD4, and βTCR followed by flow cytometric analysis. Levels of surface expression of CD69 on CXCR3^+^CCR6^-^CD4^+^ T cells, CXCR3^-^CCR6^+^CD4^+^ T cells, or CXCR3^+^CCR6^+^CD4^+^ T cells were determined by geometric mean fluorescent intensity (gMFI) of CD69 in each cell population. Data represent the mean ± SD. Significance was determined by two-tailed Student’s *t*-test (for two groups) or by one-way ANOVA with Tukey’s *post-hoc* test (for three groups). Statistically significant differences are indicated (*p < 0.05; **p < 0.01; ***p < 0.001). ns, not significant. Each symbol represents individual mouse. Number of mice used for each group is as follows: Saline, *n* = 4; WT-SR, *n* = 4; PKD1mKO-SR, *n* = 5.

Because of the reduced MHC-II surface expression on myeloid linage cells and reduced expression of cytokines and chemokines that are known to be critical for Th cell lineage development and chemotaxis in lungs of PKD1mKO repeatedly exposed to *S. rectivirgula*, we investigated whether PKD1 deletion in myeloid lineage cells affect the CD4^+^ T-cell accumulation and activation in the lungs following exposures to *S. rectivirgula*. **Figure 8B** shows that the number of CD4^+^ T cells recovered from BAL of PKD1mKO repeatedly exposed to *S. rectivirgula* for 3 weeks was substantially lower compared to that of WT. In addition, surface expression levels of an activation marker CD69 on CD4^+^ T cells were significantly reduced in PKD1mKO compared to those in WT. These results indicate that PKD1 in myeloid lineage cells contributes to influx and activation of CD4^+^ T cell in the lungs.

Chemokine receptors expressed on Th cells generally reflect the lineage of Th cells (76, 77). Th1 cells that produce IFNγ express CXCR3 but not CCR4 and CCR6. Th2 cells that produce IL-4 express CCR4 but not CXCR3 and CCR6. Th17 cells that produce IL-17 express CCR6 but not CXCR3 and CCR4. Under certain pathologic conditions, Th17 cells acquire the ability to express CXCR3 and produce both IFNγ and IL-17. These CXCR3^+^CCR6^+^ CD4^+^ T cells are called nonconventional Th1 cells or pathogenic Th1/Th17 cells (76, 77). The observed reduction in the expression of chemokines CXCL9, CXCL10, CXCL11, and CCL20 in PKD1mKO mice (compared to WT mice) in response to repeated exposures to *S. rectivirgula* predicts reduced recruitment of CXCR3^+^ Th1 cells and CCR6^+^ Th17 cells into the lungs in PKD1mKO following repeated exposures to *S. rectivirgula*. Thus, we investigated whether deletion of PKD1 in myeloid lineage cells influences accumulation of Th1 and/or Th17 cells into lungs of mice following repeated exposures to *S. rectivirgula*.

**Figure 9:**
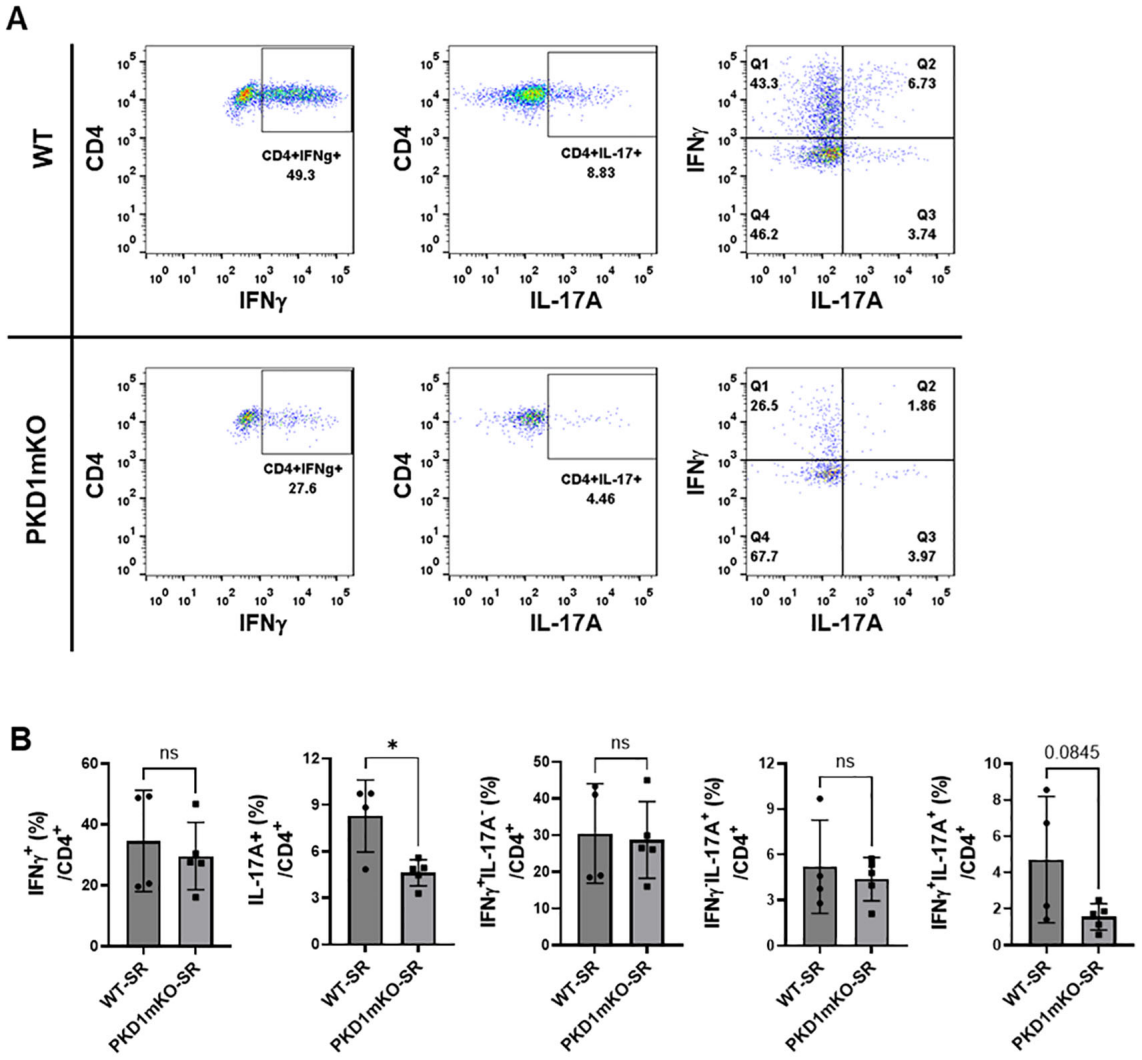
Effects of myeloid lineage cell-specific PKD1 on Th1/Th17 cell accumulation in the lungs following repeated exposures to *S. rectivirgula*. PKD1*^fl/fl^* mice (WT) and PKD1*^fl/fl^*-LyZ*^Cre^* mice (PKD1mKO) were exposed intranasally to SR (100 μg) three times per week for 3 weeks. Seventy-two hours after the last *S. rectivirgula* challenge, LICs were collected. LICs were stimulated with PMA plus ionomycin and then processed for intracellular IL-17A and IFNγ detection as described in Materials and Methods. Stained LICs were then subjected to flow cytometric analysis. **(A)** Gating of CD4^+^ T cells for IFNγ and/or IL-17A expression. **(B)** The frequency of IFNγ^+^, IL-17A^+^, IFNγ^+^IL-17A^-^ cells (Q1), IFNγ^+^IL-17A^+^ cells (Q2), or IFNγ^-^IL-17A^+^ cells (Q3) in the CD4^+^ T-cell population is expressed as % in the CD4^+^ cell population. Data represent the mean ± SD. Significance was determined by two-tailed Student’s *t*-test. Statistically significant differences are indicated (*p < 0.05). ns, not significant. Each symbol represents individual mouse. Number of mice used for each group is as follows: WT-SR, *n* = 4; PKD1mKO-SR, *n* = 5.

We analyzed CD4^+^ T cells expressing CXCR3 and/or CCR6 in the bronchoalveaolar spaces (**Figure 8A**) and CD4^+^ T cells expressing IFNγ and/or IL-17A in the lung interstitium (**Figure 9A**). As shown in **Figure 8C**, compared to WT mice repeatedly exposed to *S. rectivirgula* for 3 weeks, the frequency of CXCR3^+^CCR6^-^ cells (Th1 cells) among CD4^+^ T cells was significantly increased in the bronchoalveaolar spaces of PKD1mKO mice repeatedly exposed to *S. rectivirgula* for 3 weeks. However, the number of CXCR3^+^CCR6^-^CD4^+^ T cells recovered from BAL was not significantly different between WT and PKD1mKO. Surface expression levels of CD69 on CXCR3^+^CCR6^-^CD4^+^ T cells were significantly lower in PKD1mKO than in WT. Intracellular staining of LICs for IFNγ and IL-17A showed that frequencies of IFNγ^+^IL-17A^-^ cells (Th1 cells) among CD4^+^ T cells isolated from lung interstitium were comparable between WT and PKD1mKO exposed to *S. rectivirgula* for 3 weeks (**Figure 9B**). Judged by the lower levels of lung-infiltrated leukocytes and the lower frequency of CD45^+^ cells isolated from lung interstitium (**Figure 5B**; **Supplementary Figure 6**; **Supplementary Table 2**), the actual number of IFNγ^+^IL-17A^-^CD4^+^ T cells in lung interstitium of PKD1mKO exposed to *S. rectivirgula* for 3 weeks may be lower compared to that in WT exposed to *S. rectivirgula* for 3 weeks. These results indicate that PKD1 deletion in myeloid lineage cells may not significantly affect the CXCR3^+^CCR6^-^ classical Th1 cell infiltration into the bronchial airway and lung interstitium in the lung following repeated exposures to *S. rectivirgula*.

Frequency and number of CXCR3^-^CCR6^+^ cells (Th17 cells) among CD4^+^ T cells in the bronchoalveaolar spaces of PKD1mKO repeatedly exposed to *S. rectivirgula* for 3 weeks was significantly lower than those in WT (**Figure 8D**). Surface expression levels of CD69 on CXCR3^-^CCR6^+^CD4^+^ T cells were comparable between WT and PKD1mKO exposed to *S. rectivirgula* for 3 weeks. Frequencies of IFNγ^-^IL-17A^+^ cells (Th17 cells) among CD4^+^ T cells isolated from lung interstitium were also comparable between WT and PKD1mKO exposed to *S. rectivirgula* for 3 weeks (**Figure 9B**). Judged by the lower levels of lung-infiltrated leukocytes and the lower frequency of CD45^+^ cells isolated from lung interstitium (**Figure 5B**; **Supplementary Figure 6**; **Supplementary Table 2**), the actual number of IFNγ^-^IL-17A^+^CD4^+^ T cells in lung interstitium of PKD1mKO exposed to *S. rectivirgula* for 3 weeks may be considerably lower compared to that in WT exposed to *S. rectivirgula* for 3 weeks. These results indicate that CXCR3^-^CCR6^+^ classical Th17 cells are a minor Th cell subpopulation in both bronchoalveaolar spaces and lung interstitium after 3-week exposures to *S. rectivirgula* and that PKD1 deletion in myeloid lineage cells may significantly affect the CXCR3^-^CCR6^+^ classical Th17 cell accumulation in the bronchoalveaolar spaces and lung interstitium following repeated exposures to *S. rectivirgula*.

As shown in **Figure 8E**, both the frequency and number of CXCR3^+^CCR6^+^CD4^+^ T cells (which is known as nonconventional Th1 cell or pathogenic Th1/Th17 cells) in the bronchoalveaolar spaces were significantly reduced in PKD1mKO mice repeatedly exposed to SR for 3 weeks compared to those in WT. In addition, surface expression levels of CD69 on CXCR3^+^CCR6^+^CD4^+^ T cells in the bronchoalveaolar spaces of PKD1mKO exposed to *S. rectivirgula* for 3 weeks were significantly lower than CD69 levels on CXCR3^+^CCR6^+^CD4^+^ T cells from WT mice exposed to *S. rectivirgula* for 3 weeks. Frequencies of IFNγ^+^IL-17A^+^ cells (nonconventional Th1 cells) among CD4^+^ T cells isolated from the lung interstitium of PKD1mKO exposed to *S. rectivirgula* for 3 weeks were substantially, but not significantly (*p* = 0.845), lower than those of WT exposed to *S. rectivirgula* for 3 weeks (**Figure 9B**). Judged by the lower levels of lung-infiltrated leukocytes and the lower frequency of CD45^+^ cells isolated from lung interstitium (**Figure 5B**; **Supplementary Figure 6**; **Supplementary Table 2**), the actual number of IFNγ^+^IL-17A^+^CD4^+^ T cells in the lung interstitium of PKD1mKO exposed to *S. rectivirgula* for 3 weeks may be significantly lower compared to that in WT exposed to *S. rectivirgula* for 3 weeks. These results demonstrate that CXCR3^+^CCR6^+^ nonconventional Th1 cells are the major Th cell subpopulation in the bronchoalveaolar spaces, while it is the minor Th cell subpopulation in lung interstitium, following 3-week exposures to *S. rectivirgula* and that PKD1 in myeloid lineage cells contribute significantly to accumulation and activation of CXCR3^+^CCR6^+^ nonconventional Th1 cells in the lung following repeated exposures to *S. rectivirgula*. Taken together, our results indicate that PKD1 in myeloid lineage cells contributes to the accumulation of pathogenic Th1/Th17 cells in the lungs in HP caused by repeated exposures to *S. rectivirgula*.

## Discussion

We demonstrated in the current study that although it is dispensable for the initial immediate pulmonary inflammatory responses and HP development caused by *S. rectivirgula*, PKD1 in myeloid lineage cells contributes significantly to the alveolitis, proinflammatory cytokine and chemokine production, and accumulation of CXCR3^+^CCR6^+^ nonconventional Th1 cells in the lung during the development of HP caused by *S. rectivirgula*.

We have previously reported that the HP-inciting agent *S. rectivirgula* induces activation of PKD1 in alveolar epithelial cells, alveolar macrophages, and neutrophils via an MyD88-dependent pathway and that PKD1 contributes significantly to the acute and chronic pulmonary proinflammatory responses leading to Th1- and Th17-promoting milieu in the lungs, and development of HP caused by repeated *S. rectivirgula* inhalation (43, 57). Previous studies have indicated the importance of neutrophils for the development of HP (59). Neutrophils infiltrated into the lung are the major producers of IFNγ after *S. rectivirgula* inhalation in acute HP, and IFNγ produced by innate immune cells is sufficient to induce HP in this model (63). Resident macrophages are critical for the detection of antigens and the production of cytokines and chemokines in the acute phase of HP (9, 58). Neutrophils and macrophages play a preponderant role in initiation and progression of HP, and PKD1 is essential for the initial expression of various proinflammatory mediators through the TLR/MyD88-signaling pathway activated by *S. rectivirgula* in these cells (43, 57). Therefore, we investigated the contribution of PKD1 activation in these cells to the development of HP caused by *S. rectivirgula* using a conditional knockout mouse in which PKD1 in myeloid lineage cells has been deleted. In the previous study using tamoxifen-induced PKD1-insuficient mice (systemic PKD1-knockout mice), we showed that the initial mRNA expression of TNFα, IL-6, IL-12, CCL2, CCL3, CCL5, CXCL2, and CXCL5 in the lungs at 1 h after exposure to *S. rectivirgula* and CXCL2 protein levels in BALF at 6 h after exposure to *S. rectivirgula* are significantly inhibited in the absence of PKD1 (57). In contrast to what was observed in PKD1-insufficient mice, the initial mRNA expression levels of TNFα, IL-6, CCL2, CCL4, CXCL1, CXCL2, and CXCL10 in the lung at 2 h and 6 h post-*S. rectivirgula* inhalation were not significantly different between WT mice and PKD1mKO. Protein levels of CCL2, CXCL1, and CXCL2 in BALF were also not significantly different between WT mice and PKD1mKO at 6 h post-*S. rectivirgula* inhalation. However, levels of these proinflammatory cytokines and chemokines in the lungs were significantly lower in PKD1mKO compared to WT at 24 h after the *S. rectivirgula* exposure, indicating an essential role of PKD1 in proinflammatory cytokine and chemokine expression in newly lung-infiltrated neutrophils fueling the higher leukocyte infiltration into the lungs. Reflecting the significant reduction in expression of chemokines (CCL3, CCL4, CXCL1, CXCL2, and CXCL10) in the lung, the levels of alveolitis in PKD1mKO at 24 h after the *S. rectivirgula* exposure was also significantly reduced compared to the levels of alveolitis in WT. At 24 h after the single exposure to *S. rectivirgula*, the levels of alveolitis in PKD1mKO were similar to the levels of alveolitis in PKD1-insufficient mice previously reported (the number of total cells recovered from the BALF from *S. rectivirgula*-exposed PKD1-insufficient mice and PKD1mKO were approximately 33% and 35%, respectively, of those in *S. rectivirgula*-exposed WT mice) (57). Since PKD1 deficiency does not affect neutrophil’s migration ability toward CXCL2, the observed significant reduction in proinflammatory cytokines and chemokines in the lungs of PKD1mKO at 24 h after the *S. rectivirgula* exposure are presumably due to both reduced expression of these cytokines and chemokines in myeloid cells (residential alveolar macrophages and newly infiltrated neutrophils) and subsequently reduced influx of neutrophils and other leukocytes into the lungs. Taken together, our results indicate that although it is dispensable for the initial proinflammatory responses in the lungs immediately after the *S. rectivirgula* exposure (the period when other types of lung-resident cells like alveolar epithelial cells are predominant responders and/or when PKD1-independent signaling pathways, which are yet to be identified, are responsible for cytokine and chemokine expression in myeloid lineage cells), PKD1 in myeloid lineage cells plays an indispensable role for the acute pulmonary proinflammatory responses following one-time exposure to *S. rectivirgula*.

Neutrophil chemoattractant CXCL8 is increased in BALF of HP patients and contribute to the development of the HP (16, 78). Human alveolar macrophages and respiratory epithelial cells can release CXCL8 in response to organic antigens, including *S. rectivirgula* (16, 78, 79). *S. rectivirgula* can induce activation of PKDs in an IRAK4-dependent manner in human macrophages (**Supplementary Figure 11A**). This *S. rectivirgula*-mediated activation of PKDs contributes significantly to the expression of TNFα, IL-6, and CXCL8 in human macrophages (**Supplementary Figure 11B**). These suggest a possibility that PKD family proteins, presumably PKD1, may contribute to the pulmonary inflammation and development of HP in human. Though mice lack the gene for CXCL8, they express the CXCL8 receptor CXCR2 and murine CXCR2 can bind to human CXCL8, as well as mouse CXCL1 and CXCL2 (80, 81). A study with the lung-targeted hCXCL8 transgenic mice showed that hCXCL8 expression in the mouse bronchial epithelium mediates neutrophilia in the airways, with enhanced maturation and activation of neutrophils, and causes lung remodeling, with inflammation, mucus hypersecretion, fibrosis, and leaky tight junctions, which results in reduced lung function (82). Future investigations using myeloid lineage cell-targeted hCXCL8 transgenic mice or other humanized mice system would help our understanding for pathogenesis of HP in human.

The initial proinflammatory cytokine and chemokine milieu and degree of leukocyte influx affect the outcome of HP progress. Similar to what was observed in PKD1-insufficient mice, repeated exposures of PKD1mKO to *S. rectivirgula* resulted in significantly reduced alveolitis and suppressed granuloma formation in the lungs compared with those in WT mice. However, the reduction in alveolitis in PKD1mKO appears less than that in PKD1-insufficient mice previously reported. The number of total cells recovered from the BALF from PKD1-insufficient mice repeatedly exposed to *S. rectivirgula* for 5 weeks was approximately 30% of that in PKD1-sufficient mice (57). Compared to that, the number of total cells recovered from the BALF from PKD1mKO repeatedly exposed to *S. rectivirgula* for 3 weeks were approximately 62% of those in *S. rectivirgula*-exposed WT mice. These findings indicate that in addition to PKD1 activation in myeloid lineage cells, PKD1 activation in other types of lung-resident and infiltrated cells in response to *S. rectivirgula* contribute significantly to the development and progression of HP. A residual phenotype in PKD1mKO mice also suggests a possibility of the presence of additional pathways compensating for the loss of PKD1, which has yet to be identified. In addition, an imperfect gene deletion efficiency of the Lyz*^Cre^* strain should be factored in. A deletion efficiency of 83%–98% in mature macrophages, near 100% in granulocytes, and 16% in CD11c^+^ DCs had been reported (64). A more comprehensive analysis of the efficiency and specificity of the Lyz*^Cre^* strain showed approximately 70%–80% deletion in neutrophils and approximately 90%–100% deletion in mature macrophage populations, including alveolar macrophages and peritoneal macrophages. In contrast, it showed only approximately 40% deletion in monocytes and inflammatory monocytes (83). Thus, though PKD1 in PKD1mKO may efficiently be deleted in lung-resident macrophages and neutrophils, it may not be deleted to show sufficient functional defects in monocytes and monocyte-derived macrophages (especially in inflammatory tissues), which turn over quickly. Nonetheless, our results also demonstrate that although it is dispensable for HP development, PKD1 activation in myeloid lineage cells in response to *S. rectivirgula* is a significant contributor to the progression of HP caused by repeated inhalation of *S. rectivirgula*.

MHC-II is constitutively expressed in professional antigen-presenting cells (APCs), such as dendritic cells and macrophages, and critical for the activation of Th cells via presenting extracellular antigen-derived peptides to T-cell receptor on Th cells (84). Previous studies have shown that under certain disease conditions like infection, chronic inflammation, and autoimmune diseases, expression of MHC-II can also be induced in certain non-APCs as well as APCs and that non-APCs acquired the expression of MHC-II function as APCs (60, 84–86). We previously reported that MHC-II surface expression on macrophages and PMNs in the lungs is increased following repeated exposures to *S. rectivirgula* and PKD1 influences these increased MHC-II surface expression (57). Similar to this previous finding using PKD1-insufficient mice, PKD1 deletion in myeloid-lineage cells also resulted in significantly suppressed surface expression of MHC-II on myeloid lineage cells accumulated in the lungs following repeated exposures to *S. rectivirgula*. We currently do not know the mechanisms by which PKD1 in myeloid lineage cells contribute to the MHC-II surface expression on myeloid lineage cells in the lungs following repeated intranasal exposures to *S. rectivirgula*. This may be largely due to the overall reduction in inflammatory cytokines that we observed in the PKD1mKO lungs, especially IFNγ that functions as a potent inducer of MHC-II expression on both professional and non-professional APCs (84, 86, 87). Several other possible mechanisms for PKD1 involvement in increased MHC-II surface expression in myeloid lineage cells in HP, such as transportation of MHC-II containing cargo from the trans-Golgi network to the cell surface and MyD88-dependent TLR-mediated downregulation of MARCH1, also exist and cannot be completely ruled out. The mechanisms by which PKD1 regulates MHC-II expression in myeloid lineage cells following repeated exposures to *S. rectivirgula* are yet to be delineated.

As HP progresses, the initial neutrophilic alveolitis becomes progressively more lymphocytic. Pulmonary cytokine and chemokine milieu promoting Th1 cell and Th17 cell influx and differentiation are critical to progression of HP and the disease severity (33, 72, 73). IFNγ and IL-17A have been established as the critical cytokines in HP (33, 73, 88, 89). IL-17, IFNγ, and CXCL10 are significantly increased in the serum and BALF of the chronic bird-related HP patients after the inhalation provocation test (90). Alveolar macrophages from patients with HP express higher mRNA levels of CXCL9 and CXCL10 than control alveolar macrophages and striking levels of these CXCR3 ligands are detected in the BALF from HP patients (91). Previous studies indicated that IFNγ mediates expression and secretion of chemokines CXCL9, CXCL10, and CXCL11 in macrophages, resulting in the recruitment of CXCR3 bearing CD4^+^ Th cells and CD8^+^ Tc cells into the lung and granuloma formation in humans and mice (70, 91). IL-17A has been shown to be produced by γδ T cells, CD4^+^ Th17 cells, neutrophils, and monocytes/macrophages in HP (89, 92–95). TNFα induces IL-17 production by Th17 cells and is involved in Th17 differentiation by promoting IL-6 and IL-1β production in monocytes (96, 97). IL-23 and TNFα act as survival signals for Th17 (98, 99). TNFα and IL-1β can induce CCL20, which can be produced by airway epithelia and neutrophils and attract CCR6 bearing Th17 cells to the site of inflammation (75, 100, 101). IL-17A and Th17 cells participate in granuloma formation and fibrosis in HP and sarcoidosis (73, 95, 102). We previously reported that expression levels of IL-17A in the lung following repeated exposures to *S. rectivirgula* are almost completely, if not completely, ablated in PKD1-insufficient mice (57). In contrast, deletion of PKD1 in myeloid lineage cells resulted in significant, but partial reduction in cytokines IFNγ, TNFα, IL-1β, IL-6, IL-17A, IL-12, and IL-23 and chemokines CXCL9, CXCL10, CXCL11, and CCL20 in the lung of mice following repeated exposures to *S. rectivirgula*. These results indicate that both PKD1 in myeloid lineage cells and PKD1 in non-myeloid lineage cells, including alveolar epithelial cells, contribute significantly to the optimal Th17-related cytokine and chemokine production during HP caused by *S. rectivirgula*. Nonetheless, reduction in these cytokines and chemokines in the lung of PKD1mKO following repeated exposures to *S. rectivirgula* was also reflected in reduced number of CD4^+^ T cells and alterations in Th cell subpopulation composition in the airways. In the airways of WT repeatedly exposed to *S. rectivirgula*, CXCR3^+^ conventional Th1 cells were the major population (approximately 30%) followed by CXCR3^+^CCR6^+^ nonconventional Th1 cells (approximately 22%) and CCR6^+^ Th17 cells (approximately 15%). Reduction of Th cells in PKD1mKO repeatedly exposed to *S. rectivirgula* (compared to those in WT) is in both CXCR3^+^CCR6^+^ nonconventional Th1 cells and CCR6^+^ conventional Th17 cells. In addition, surface expression levels of CD69 on nonconventional Th1 cells were significantly suppressed in PKD1mKO, indicating that PKD1 activation in myeloid lineage cells preferentially affects the lung accumulation and activation of CXCR3^+^CCR6^+^ nonconventional Th1 cells by contributing to the expression of Th1- and Th17-related cytokines and chemokines and the surface expression of MHC-II in myeloid lineage cells. Although our results show that PKD1 in myeloid lineage cells contributes substantially to the Th1/Th17 responses in the lungs in mice that repeatedly inhaled *S. rectivirgula* for 3 weeks, it is still unknown whether these responses differ in the induction phase of Th cell development or in the amplification of the Th response after infiltration into the lungs. Further investigations to address this issue in the future are warranted.

The local cytokine environment affects naïve Th cell differentiation to the distinct Th effector lineage cells and trans-differentiation of a specific Th lineage cells to the other Th lineage (103–106). Th17 cells can transdifferentiate to Th1 cells in the presence of IL-1β, IL-23, IL-12, and/or TNFα, and Th1 cells can transdifferentiate to Th17 cells in the presence of TGFβ, IL-6, IL-1β, and IL-23 (105–107). These Th17-derived Th1 cells and Th1-derived Th17 cells are called with various names like nonconventional Th1, non-classic Th1, Th1*, Th17/Th1, Th1/Th17, pathogenic Th17, or non-classical proinflammatory Th17.1. They exhibit characteristics of both Th1 and Th17 cells: they express both CXCR3 and CCR6, express T-bet and RORC, and secrete IFNγ and IL-17A (77, 105–109). These nonconventional Th1 cells have been known to be more aggressive and more pathogenic than Th17 cells and linked to various autoimmune and inflammatory diseases such as Crohn’s disease, juvenile idiopathic arthritis, rheumatoid arthritis, multiple sclerosis, and pulmonary *Mycobacterium tuberculosis* (105, 108, 109). Nonconventional Th1 cells in HP have not been well studied. Considering the role of nonconventional Th1 cells in other inflammatory diseases and the possible correlation of ameliorated HP and reduced number and activation of nonconventional Th1 cells observed in our study, it is important and necessary to investigate in the future that any specific subset of myeloid lineage cells play a critical role in the expansion of nonconventional Th1 cells, whether nonconventional Th1 cells are more pathogenic than conventional Th1 cells or Th17 cells in HP, and whether nonconventional Th1 cells can be an effective target for HP therapy.

In summary, using conditional knockout mice that have PKD1 deleted in myeloid lineage cells, we found that PKD1 activation in myeloid lineage cells in response to the HP-inciting antigen *S. rectivirgula* contributes significantly to acute lung injury, including neutrophilic alveolitis and pulmonary inflammation by upregulating the expression of IL-1β, IL-6, TNFα, IFNγ, CCL2, CCL3, CCL4, CXCL1, CXCL2, and CXCL10. PKD1 in myeloid lineage cells also contributes to the development of subacute HP following repeated exposures to *S. rectivirgula*. PKD1 in myeloid linage cells contributes to the increased expression of TNFα, IFNγ, IL-1β, IL-12, IL-17A, IL-23, CXCL9, CXCL10, CXCL11, and CCL20, resulting in CXCR3^+^CCR6^+^ nonconventional Th1 cell accumulation and activation in the lung, alveolitis, and granuloma formation following repeated exposures to *S. rectivirgula*. Taken together, our findings imply that TLR/MyD88-dependent PKD1 activation in myeloid linage cells may play a significant role in pulmonary proinflammatory responses and the development of HP caused by *S. rectivirgula* inhalation.

## Data Availability

The original contributions presented in the study are included in the article/**Supplementary Material**, further inquiries can be directed to the corresponding author/s.
