# Corrigendum: Protein kinase D1 in myeloid lineage cells contributes to the accumulation of CXCR3^+^CCR6^+^ nonconventional Th1 cells in the lungs and potentiates hypersensitivity pneumonitis caused by *S. rectivirgula*

**DOI:** 10.3389/fimmu.2024.1513635

**Published:** 2024-11-05

**Authors:** John D. Snyder, Tae Won Yoon, Sangmin Lee, Priyanka Halder, Elizabeth Ann Fitzpatrick, Ae-Kyung Yi

**Affiliations:** ^1^ Integrated Biomedical Science Graduate Program, The University of Tennessee Health Science Center, Memphis, TN, United States; ^2^ Department of Microbiology, Immunology and Biochemistry, The University of Tennessee Health Science Center, Memphis, TN, United States

**Keywords:** protein kinase D1, cytokines/chemokines, inflammation, alveolitis, *Saccharopolyspora rectivirgula*, hypersensitivity pneumonitis, toll-like receptor signaling

In the published article, there was an error in **Figure 2** as published. The panel numbers (A, B, C) are missing. The corrected **Figure 2** and its caption appear below.

**Figure 2:**
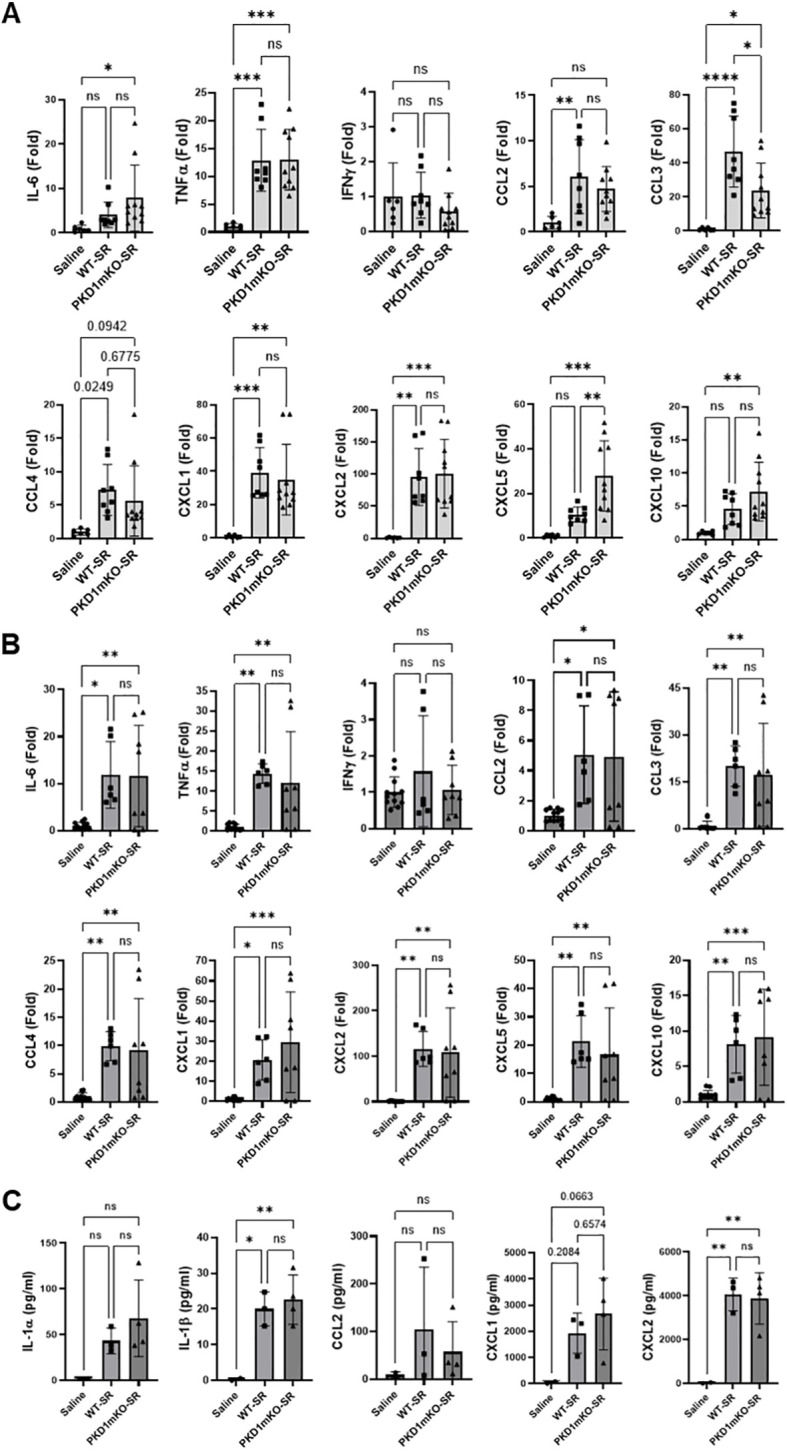
PKD1 in myeloid lineage cells is dispensable for the initial cytokine and chemokine expression in the lungs of mice in response to *S. rectivirgula* inhalation. PKD1*^fl/fl^* mice (WT) and PKD1*^fl/fl^*-LyZ*^Cre^* mice (PKD1mKO) were exposed intranasally to saline or *S. rectivirgula* (100 μg) for 2 h **(A)** or 6 h **(B, C)**. **(A, B)** Total RNA was purified from lung lobes isolated from each individual mouse and reverse transcribed, and then mRNA levels of the indicated genes were analyzed in duplicate by real-time qPCR using SYBR Green Assay. The data on genes that were differentially expressed were normalized to the expression of the housekeeping gene [Actin for panel **(A)** and GAPDH for panel **(B)**]. Fold change comparing *S. rectivirgula*-exposed WT mice and *S. rectivirgula*-exposed PKD1mKO mice to control saline-exposed mice were calculated by comparative quantification algorithm-delta delta Ct method (fold difference = 2^−ΔΔCt^). Data represent the mean (Fold) ± SD. **(C)** Bronchoalveolar lavage (BAL) was performed. Levels of the indicated cytokines and chemokines in BAL fluid were detected by multiplex sandwich assay. Data present the mean concentration (pg/mL) ± SD. Number of mice used for each group is as follows: Saline, *n* = 2 to 6; WT-SR, *n* = 3 to 4; PKD1mKO-SR, *n* = 4 to 5. Statistically significant difference determined by one-way ANOVA with Tukey’s *post-hoc* test is indicated (*p < 0.05; **p < 0.01; ***p < 0.001; ****p < 0.0001). ns, not significant.

The authors apologize for this error and state that this does not change the scientific conclusions of the article in any way. The original article has been updated.

