# Supplementary material for: Protein kinase D1 in myeloid lineage cells contributes to the accumulation of CXCR3+CCR6+ nonconventional Th1 cells in the lungs and potentiates hypersensitivity pneumonitis caused by S. rectivirgula
Source: Front Immunol. 2024 Oct 11;15:1403155. doi: 10.3389/fimmu.2024.1403155 (PMC11502317; doi:10.3389/fimmu.2024.1403155)
Supplement: Supplementary file 1 [file DataSheet1.pdf]

Protein kinase D1 in myeloid lineage cells contributes to the accumulation of CXCR3⁺CCR6⁺ nonconventional Th1 cells in the lungs and potentiates hypersensitivity pneumonitis caused by *S. rectivirgula*

John D. Snyder[1,2,†], Tae Won Yoon[1,2,†], Sangmin Lee[2], Priyanka Halder[2], Elizabeth A. Fitzpatrick [1,2], and Ae-Kyung Yi [1,2,*]

[1]Integrated Biomedical Science Graduate Program, The University of Tennessee Health Science Center, Memphis, TN 38163, U.S.A. and [2]Department of Microbiology, Immunology and Biochemistry, The University of Tennessee Health Science Center, Memphis, TN 38163, U.S.A.

Supplemental Materials

Supplemental Table 1. List of real-time PCR primers used

Gene	Chemistry	Assay ID	Primer Sequence
IL-6	SYBR Green	Mm.PT.58.10005566	5’-AGCCAGAGTCCTTCAGAGA-3’<br>5’-TCCTTAGCCACTCCTTCTGT-3’
IL-12p40	SYBR Green	Mm.PT.58.12409997	5’-TGTCCTCAGAAGCTAACCATC-3’<br>5’-TCCAGTCCACCTCTACAACA-3’
IL-17A	SYBR Green	Mm.PT.58.6531092	5’-AGACTACCTCAACCGTTCCA-3’<br>5’-GAGCTTCCCAGATCACAGAG-3’
IL-23p19	SYBR Green		5’-AGTCCTGACTGGGCTCAGTG-3’<br>5’-CAAAGACCCGGGCAGCTATG-3’
TNFα	SYBR Green	Mm.PT.58.12575861	5’-AGACCCTCACACTCAGATCA-3’<br>5’-TCTTTGAGATCCATGCCGTTG-3’
CCL2	SYBR Green	Mm.PT.58.13906306	5’-TTCCTGATGGCACTTCTCTTG-3’<br>5’-ACCTCTGTCCGTGATGATCT-3’
CCL3	SYBR Green	Mm.PT.58.29283216	5’-CCTTGCTGTTCTTCTCTGTACC-3’<br>5’-CGATGAATTGGCGTGGAATC-3’
CCL4	SYBR Green	Mm.PT.58.5219433	5’-CTCTCTCTCCTCTTGCTCGT-3’<br>5’-GTCTCATAGTAATCCATCACAAAGC-3’
CCL5	SYBR Green	Mm.PT.58.43548565	5’-GCTCCAATCTTGCAGTCGT-3’<br>5’-CCTCTATCCTAGCTCATCTCCA-3’
CCL20	SYBR Green	Mm.PT.58.13906306	5’-CCAGCACTGAGTACATCAACT-3’<br>5’-GTATGTACGAGAGGCAACAGTC-3’
CXCL1	SYBR Green	Mm.PT.58.42076891	5’-CCAAACCGAAGTCATAGCCA-3’<br>5’-GTGCCATCAGAGCAGTCT-3’
CXCL2	SYBR Green		5’-CCAGACAGAAGTCATAGCCACT-3’<br>5’-AGGCACATCAGGTACGATCC-3’
CXCL5	SYBR Green	Mm.PT.58.29518961.g	5’-TTCTGTTGCTGTTCACGCT-3’<br>5’-ATCACCTCCAAATTAGCGATCA-3’
CXCL9	SYBR Green	Mm.PT.58.5726745	5’-CAAATCCCTCAAGACCTCAAAC-3’<br>5’-GATCTCCGTTCTTCAGTGTAGC-3’
CXCL10	SYBR Green	Mm.PT.58.43575827	5’-ATTTTCTGCCTCATCCTGCT-3’<br>5’-TGATTTCAAGCTTCCCTATGGC-3’
CXCL11	SYBR Green	Mm.PT.58.42838989	5’-GAGATGAACAGGAAGGTCACAG-3’<br>5’-GGGCCGATGCAAAGACA-3’
CXCL12	SYBR Green	Mm.PT.58.12038563	5’-GTGCCCTTCAGATTGTTGC-3’<br>5’-GACTCACACCTCTCACATCTTG-3’
CXCL16	SYBR Green	Mm.PT.56a.42520449	5’-ATCAGGTTCCAGTTGCAGTC-3’<br>5’-TTCCCATGACCAGTTCCAC-3’
GAPDH	SYBR Green	Mm.PT.39a.1	5’-AATGGTGAAGGTGTG-3’<br>5’-GTGGGTCATACTGGAACATGTAG-3’

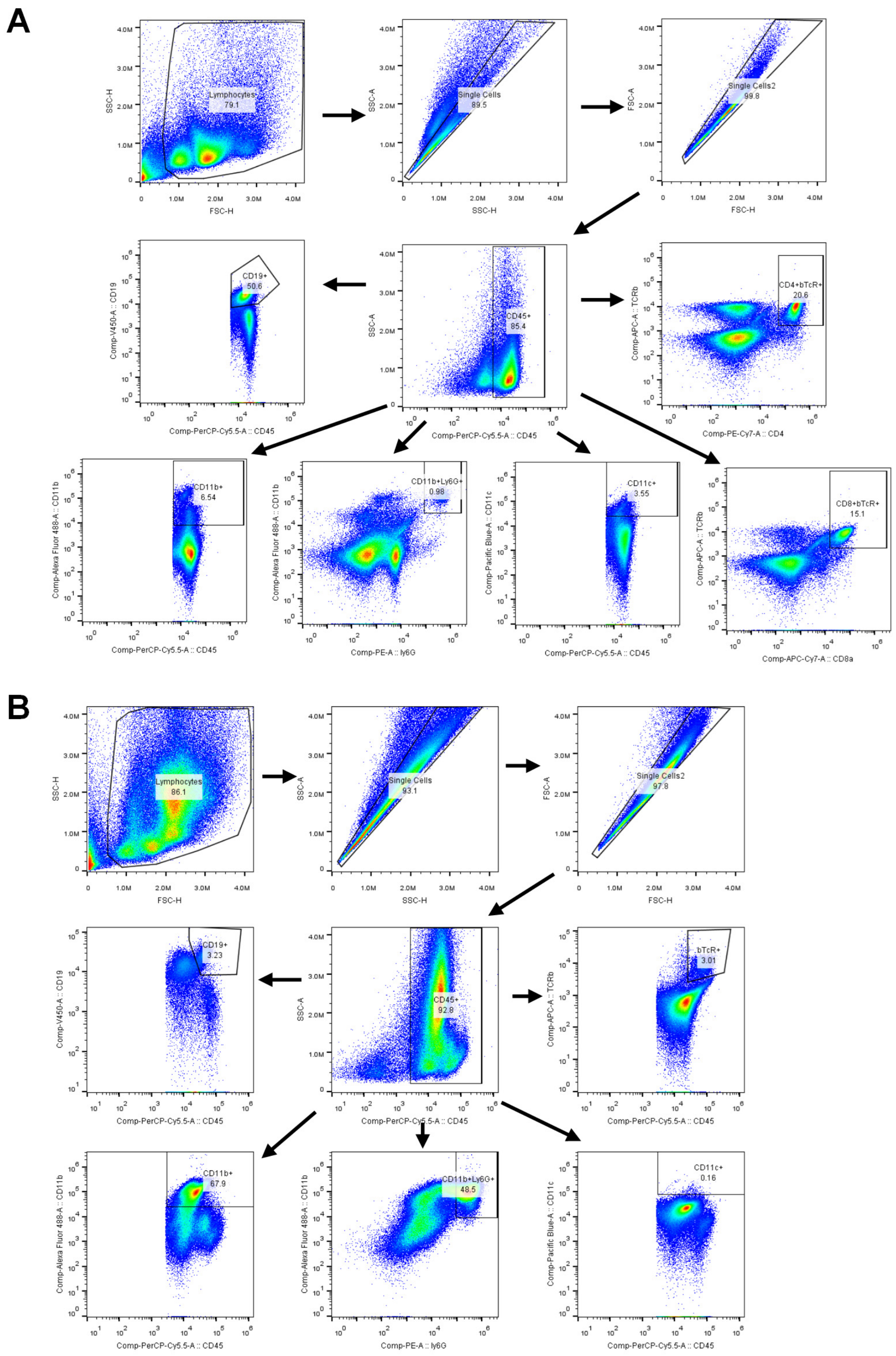

Supplemental Figure 1. Gating strategy for flowcytometric analysis. (A) Gating strategy for flowcytometric analysis of splenic cells. **(B)** Gating strategy for flowcytometric analysis of bone marrow (BM) cells.

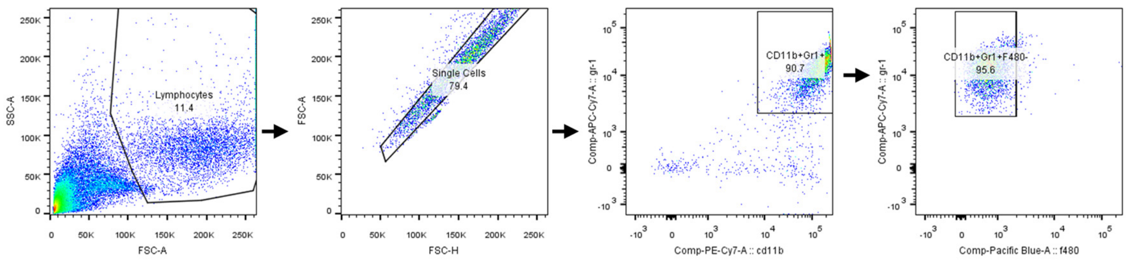

Supplemental Figure 2. Gating strategy for flowcytometric analysis of neutrophils in BAL cells. Gating strategy for flowcytometric analysis of neutrophils in BAL cells obtained from mice exposed to *S. rectivirgula* single time.

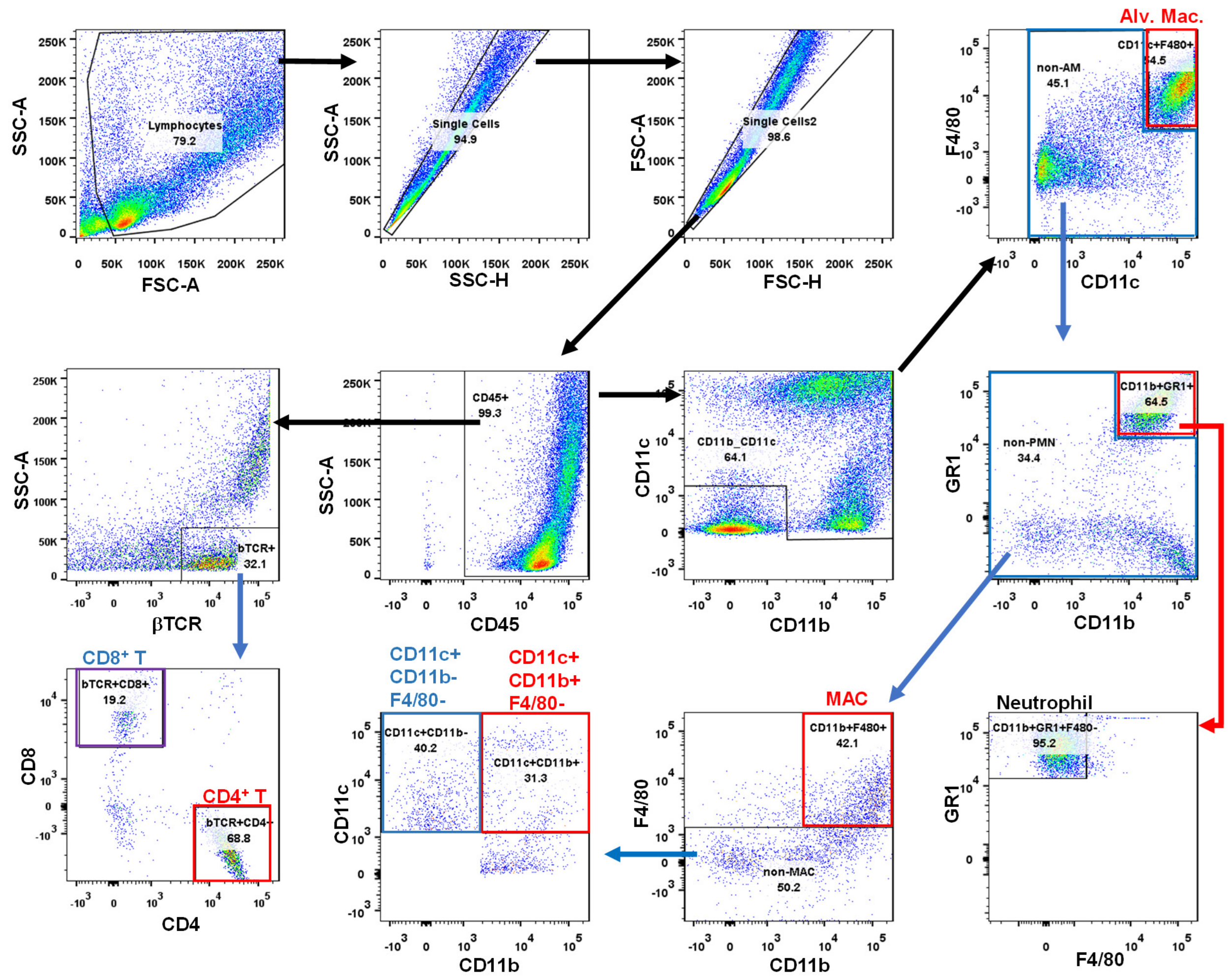

Supplemental Figure 3. Gating strategy for flowcytometric analysis of BAL cells. Gating strategy for flowcytometric analysis of BAL cells obtained at 72hr after the last *S. rectivirgula* exposure from mice exposed to *S. rectivirgula* 3 times/week for 3 weeks.

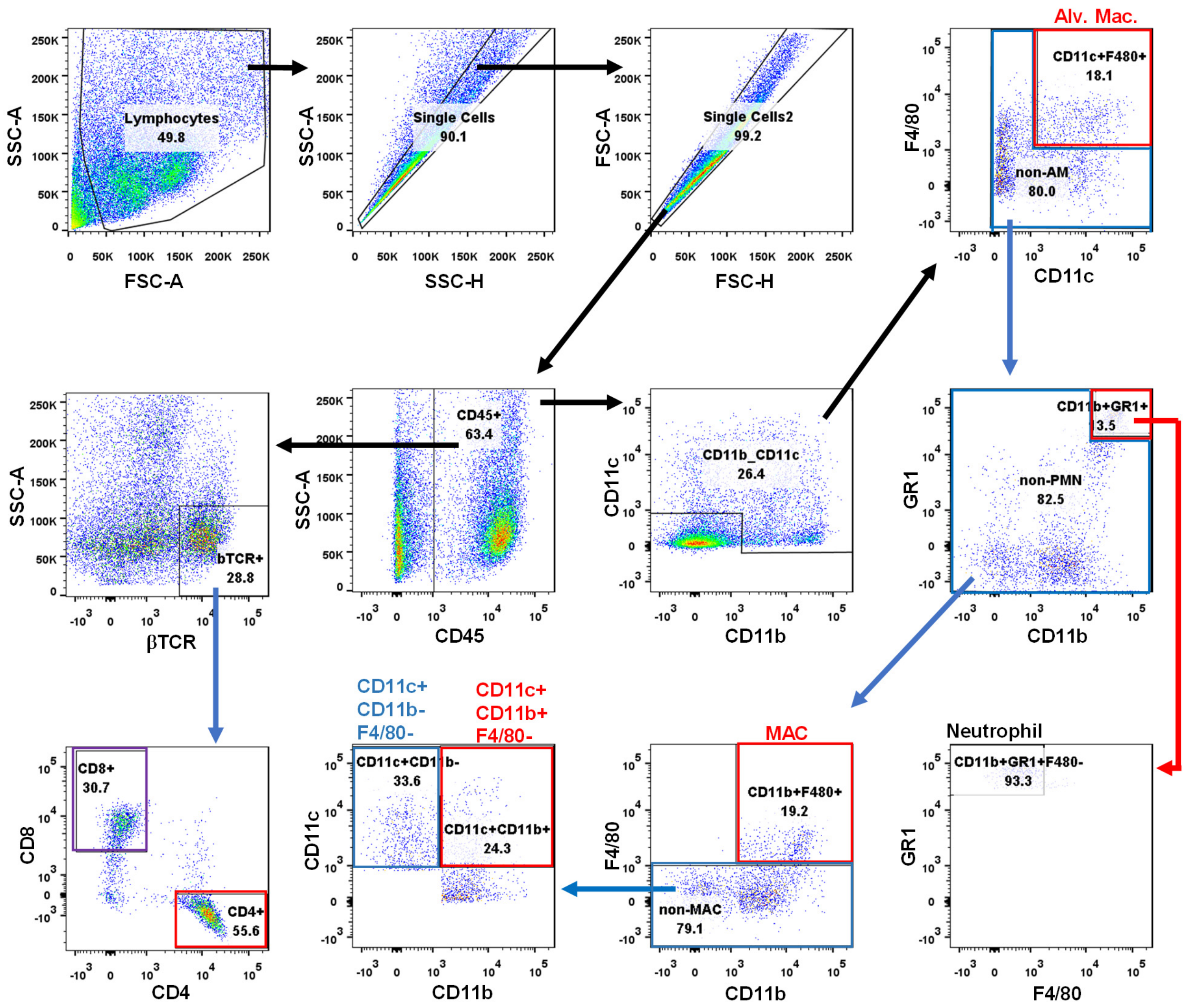

Supplemental Figure 4. Gating strategy for flowcytometric analysis of lung interstitial cells. Gating strategy for flowcytometric analysis of lung interstitial cells obtained at 72hr after the last *S. rectivirgula* exposure from mice exposed to *S. rectivirgula* 3 times/week for 3 weeks.

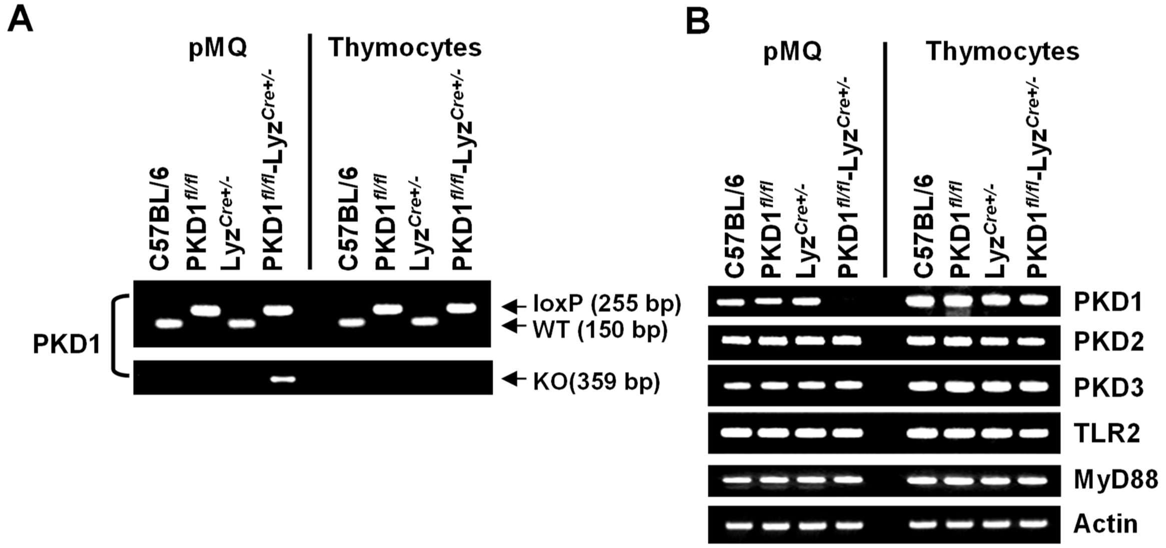

Supplemental Figure 5. Genotypic and phenotypic analysis of myeloid lineage cell-specific PKD1-deficient mice. Peritoneal macrophages (pMQ) and thymocytes were isolated. **(A)** Genomic DNA isolated from peritoneal macrophages and thymocytes were analyzed for PKD1 alleles by PCR. PCR products corresponding to PKD1 WT (151 bp), PKD1^{loxP} (255 bp), and PKD1KO (359 bp) are shown. **(B)** Total RNA was isolated from peritoneal macrophages and thymocytes. The levels of mRNA of the indicated genes were analyzed by RT-PCR.

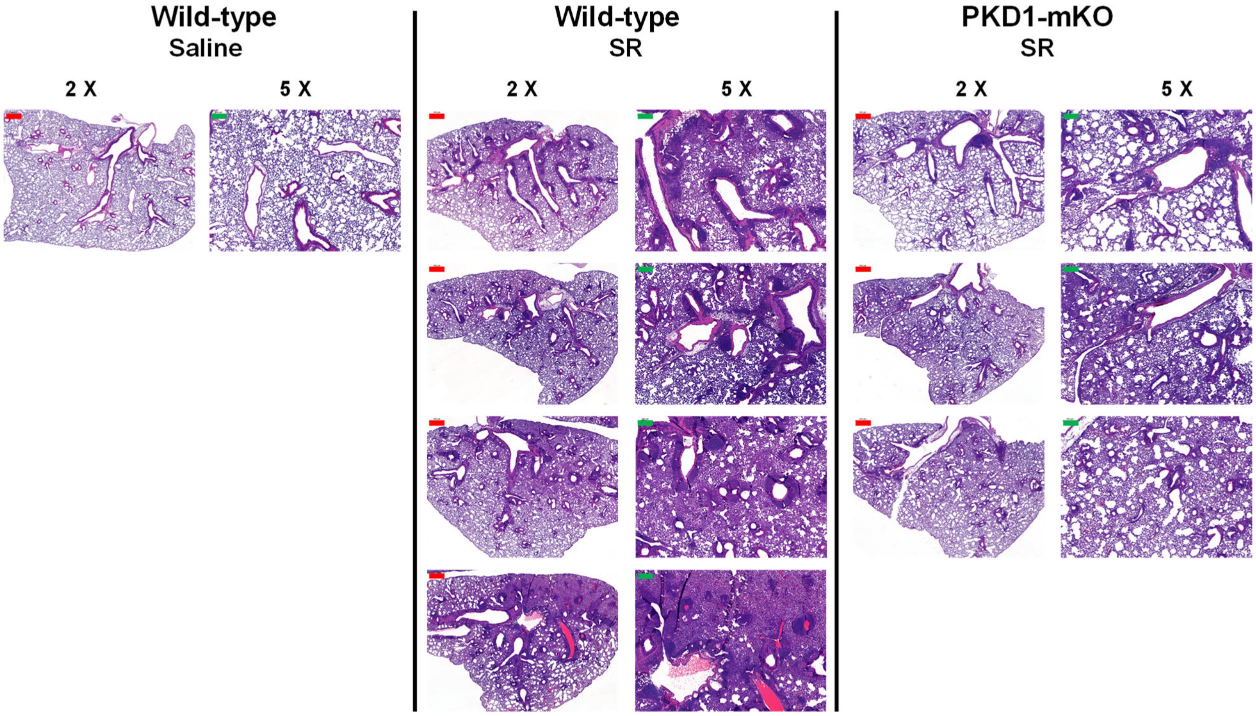

Supplemental Figure 6. Deletion of PKD1 in myeloid lineage cells results in reduced alveolitis and granuloma formation following repeated exposures to *S. rectivirgula*. PKD1$^{fl/fl}$ mice (WT) and PKD1$^{fl/fl}$-LyZ^{Cre} mice (PKD1mKO) were exposed intranasally to saline or *S. rectivirgula* (100 µg) 3 times/week for 3 weeks. Forty-eight hr after the last *S. rectivirgula* exposure, the left lung lobes removed from the mice. H&E staining of the left lung lobe sections are shown. The Aperio ScanScope®XT Slide Scanner system was used to capture whole-slide digital images. Each row represents the lung collected from an individual mouse. The images presented are 2 x magnification (scale bar = 500 µm) and 5 x magnification (scale bar = 200 µm).

Supplemental Table 2. Cellular composition of lung interstitial cells following 3-week exposures of WT and PKD1mKO mice to *S. rectivirgula*.

	$CD45^+$ (%)	AM (%)	MAC (%)	Neutrophils (%)	CD11c+ CD11b+ F4/80- (%)	CD11c+ CD11b- F4/80- (%)	$CD4^+$ T (%)	$CD8^+$ T (%)
**WT**	63.75 ± 5.06	5.57 ± 1.36	4.95 ± 3.22	5.13 ± 3.01	5.83 ± 2.53	3.52 ± 0.55	13.32 ± 1.99	10.20 ± 1.38
**PKD1mKO**	52.66 ± 3.24**	5.68 ± 1.30	4.82 ± 1.09	5.85 ± 2.66	4.36 ± 0.75	3.62 ± 0.47	13.74 ± 1.02	9.39 ± 1.57

WT (n=4) and PKD1mKO (n=5) were exposed to *S. rectivirgula* (100 μg) 3 times/wk for 3 wk and analyzed 72 h after the last exposure. The frequency of $CD45^+$ cells, alveolar macrophages (AM: $CD11c^+F4/80^+$), macrophages (MAC: $CD11b^+F4/80^+CD11c^-$), neutrophils ($CD11b^+Gr1^+F4/80^-$), $CD11c^+CD11b^-F4/80^-$ cells, $CD11c^+CD11b^+F4/80^-$ cells, $CD4^+$ T cells ($CD4^+\beta TcR^+$), and $CD8^+$ T cells ($CD8^+\beta TcR^+$) in lung interstitial cells were measured by flow cytometry followed by analysis using FlowJo software as described in Materials and Methods. Gating strategy is shown in sFig. 3A. Frequency of $CD45^+$ cell was expressed as % of interstitial lung cells. Frequency of AM, IM, neutrophil, $CD11c^+CD11b^-F4/80^-$ cells, $CD11c^+CD11b^+F4/80^-$ cells, $CD4^+$ T cell and $CD8^+$ T cell was expressed as % of $CD45^+$ cells. Values are given as mean ± SD of each group. Significance was determined by two-tailed Student's *t*-test. Statistically significant differences are indicated (**, $p < 0.01$).

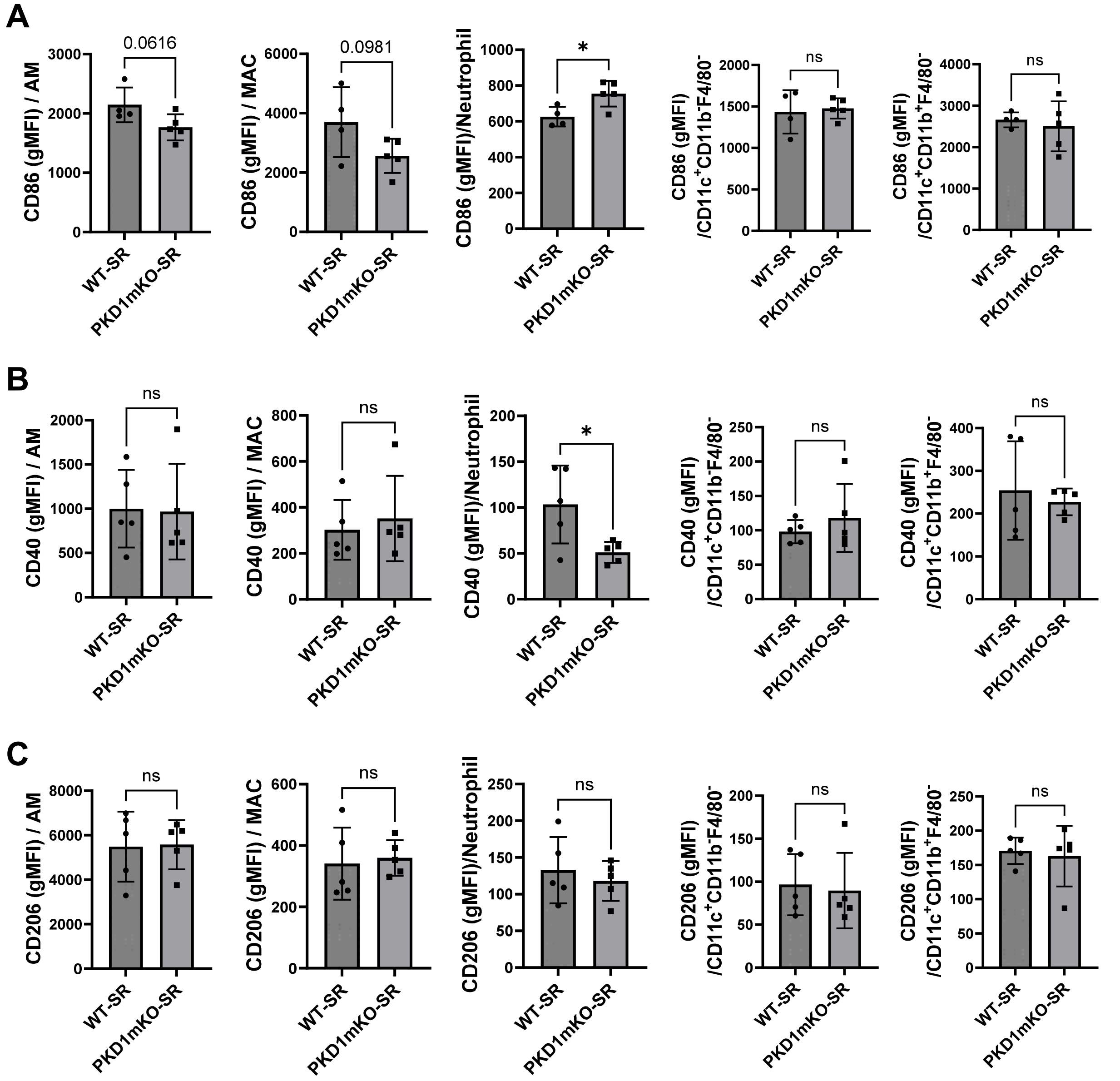

Supplemental Figure 7. Surface expression of CD86, CD40, and CD206 on myeloid lineage cells isolated from the bronchial space of WT or PKD1mKO mice exposed to *S. rectivirgula* for 3 weeks. WT (n = 4 – 5/group) or PKD1mKO mice (n = 5/group) were exposed intranasally to *S. rectivirgula* 3 times/week for 3 weeks. BAL was performed 48 h (for CD40 and CD206) or 72 h (for CD86) after the last *S. rectivirgula* exposure. BAL cells were stained with fluorochrome-conjugated Abs and then analyzed by flow cytometry and FlowJo flow software. Levels of surface expression of CD86 (Panel A), CD40 (Panel B), and CD206 (Panel C) on cells were determined by geometric mean fluorescent intensity (gMFI) of each marker in the indicated cell population. Each dot or squire represents individual mouse. Data represent the mean gMFI ± SD. Significance was determined by two-tailed Student's *t*-test. Statistically significant differences are indicated (*, $p < 0.05$). ns = not significant.

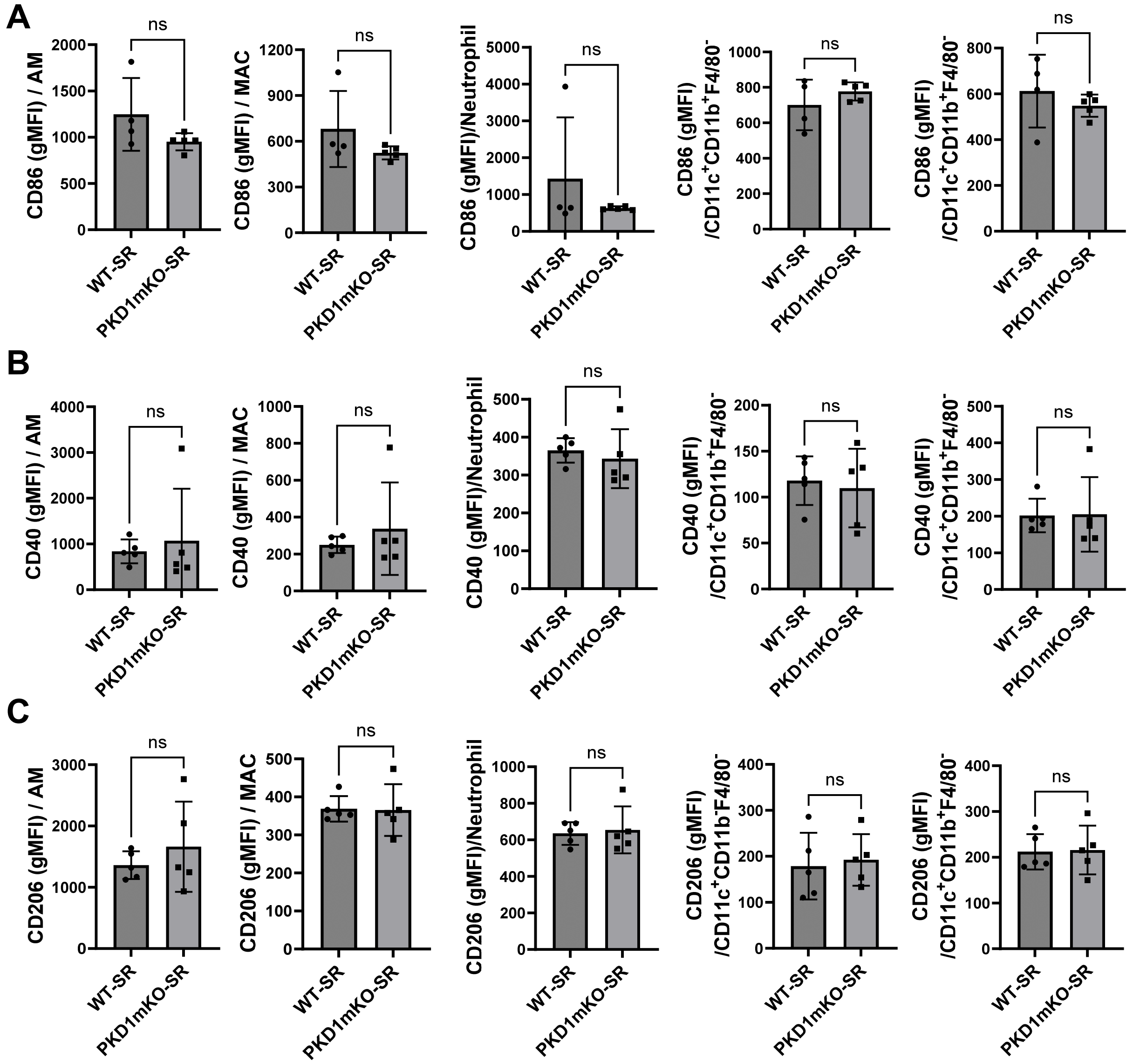

Supplemental Figure 8. Surface expression of CD86, CD40, and CD206 on myeloid lineage lung interstitial cells isolated from WT or PKD1mKO mice exposed to *S. rectivirgula* for 3 weeks. WT (n = 4 – 5/group) or PKD1mKO mice (n = 5/group) were exposed intranasally to *S. rectivirgula* 3 times/week for 3 weeks. Lung interstitial cells were isolated 48 h (for CD40 and CD206) or 72 h (for CD86) after the last *S. rectivirgula* exposure. Lung interstitial cells were stained with fluorochrome-conjugated Abs and then analyzed by flow cytometry and FlowJo flow software. Levels of surface expression of CD86 (Panel A), CD40 (Panel B), and CD206 (Panel C) on cells were determined by geometric mean fluorescent intensity (gMFI) of each marker in the indicated cell population. Each dot or squire represents individual mouse. Data present the mean gMFI ± SD. Significance was determined by two-tailed Student's *t*-test. ns = not significant.

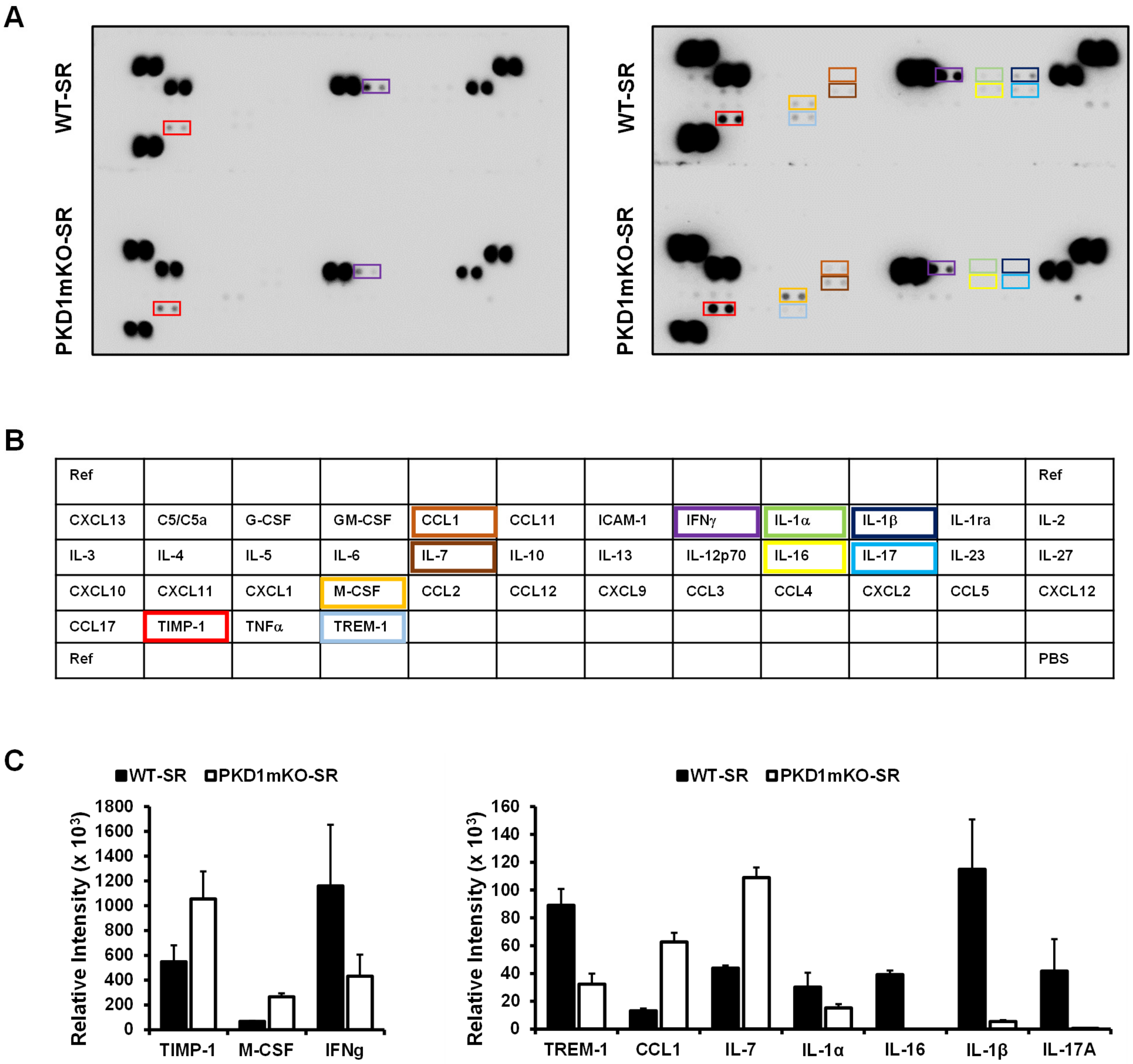

Supplemental Figure 9. Relative levels of cytokines and chemokines in the BALF following repeated exposures to *S. rectivirgula*. PKD1$^{fl/fl}$ mice (WT; n = 1) and PKD1$^{fl/fl}$-LyZ^{Cre} mice (PKD1mKO; n = 1) were exposed intranasally to *S. rectivirgula* (100 µg) 3 times/week for 3 weeks. Forty-eight hr after the last *S. rectivirgula* exposure, BAL was performed. The levels of 40 cytokines and chemokines in BALF were detected by Mouse Cytokine Array (ARY006). **(A)** Pictures of Mouse Cytokine Array scanned for short time (left panel) and long time (right panel). **(B)** Mouse Cytokine Array map with the indication of analytes that show different levels between WT-SR and PKD1mKO-SR. **(C)** Quantitation of cytokine/chemokine signals. Data present the mean signal intensity ± SD of each indicated analyte.

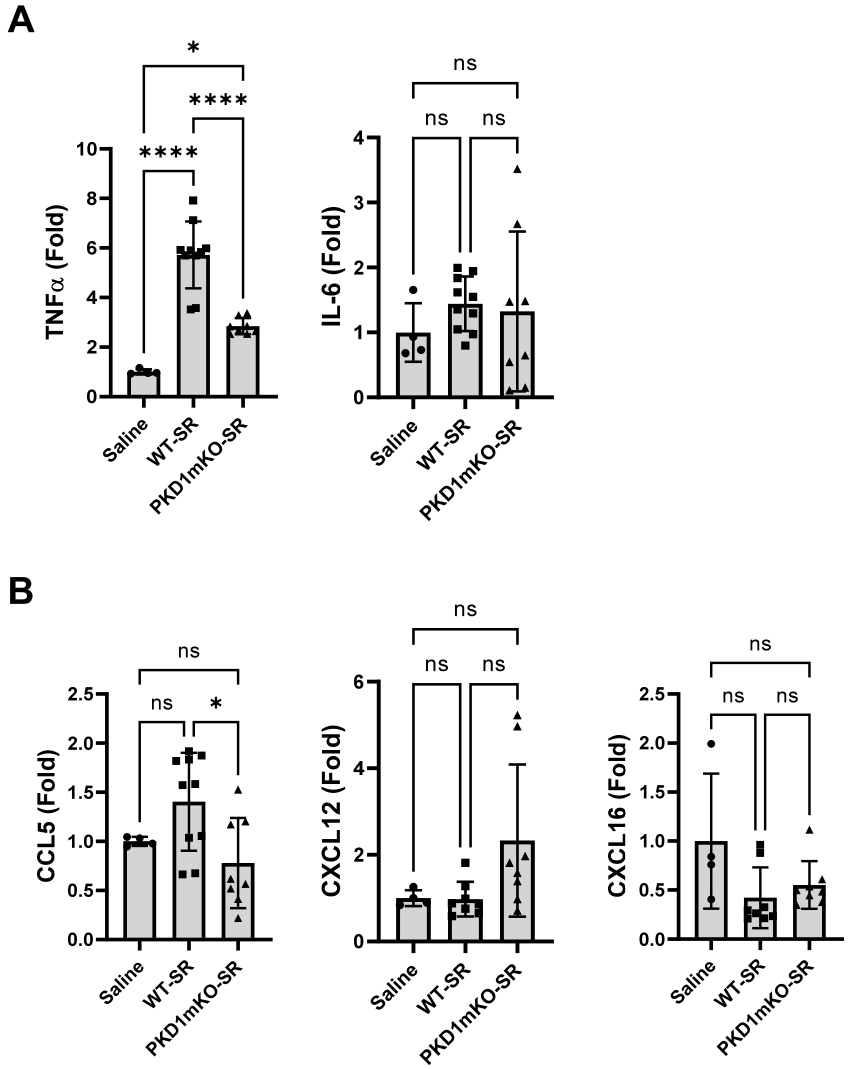

Supplemental Figure 10. Expression of cytokines and chemokines in the lung following repeated exposures to *S. rectivirgula*. PKD1$^{fl/fl}$ mice (WT) and PKD1$^{fl/fl}$-LyZ^{Cre} mice (PKD1mKO) were exposed intranasally to saline or *S. rectivirgula* (100 µg) 3 times/week for 3 weeks. Forty-eight hr after the last *S. rectivirgula* exposure, the lungs were collected, and total RNA was purified from lung lobes isolated from each individual mouse and reverse transcribed. mRNA levels of the indicated genes were analyzed in duplicates by qRT-PCR using SYBR Green Assay. The data on genes that were differentially expressed was normalized to the expression the housekeeping gene, GAPDH. Fold change comparing *S. rectivirgula*-treated exposed WT mice (WT-SR) and *S. rectivirgula*-treated PKD1mKO mice (PKD1-SR) to control mice exposed to saline were calculated by comparative quantification algorithm-delta delta Ct method (Fold difference= $2^{-\Delta\Delta Ct}$). Data represent the mean (Fold) ± SD. Significance was determined by one-way ANOVA with Tukey's post-hoc test. Statistically significant differences are indicated (*, $p < 0.05$; ****, $p < 0.0001$). ns = not significant. The number of mice used for each group is as follows: Saline, n = 1 - 2; WT-SR, n = 4 - 5; PKD1mKO-SR, n = 3 - 4.

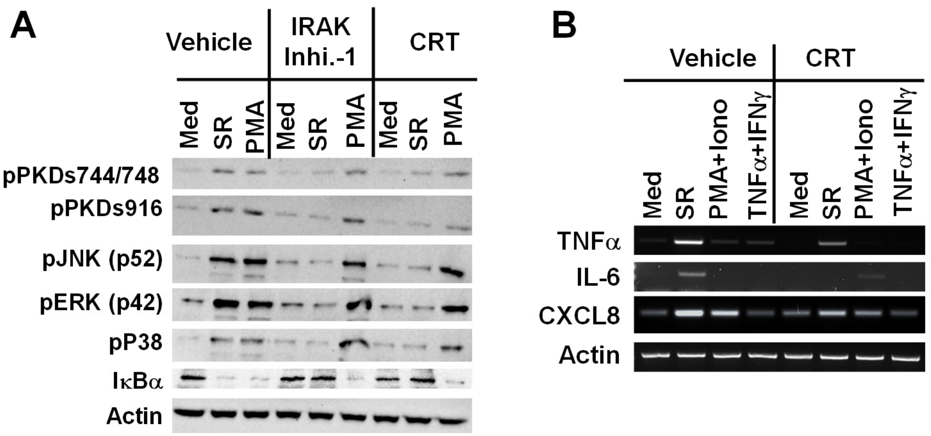

Supplemental Figure 11. *S. rectivirgula* induces IRAK4-dependent activation of PKD that contributes to the SR-mediated activation of MAPKs and NF-κB and expression of proinflammatory cytokines in human macrophages. Human monocyte cell line THP1 cells (TIB-202™; ATCC, Rockville, MD) were differentiated to macrophages as previously described (56). **A.** Differentiated THP1 cells were pretreated with vehicle (0.5% v/v DMSO), IRAK4 kinase inhibitor (IRAK inhibitor 1; 5 μM) or PKD inhibitor CRT0066101 (CRT; 10 μM) for 1 hr and then stimulated with medium (med), *S. rectivirgula* (10 μg/ml), or PMA (10 ng/ml) for 1 hr. Phosphorylation status of PKD (pPKDs744/748, pPKDs916), ERK [pERK (p42)], JNK [pJNK (p52)] and p38 (pP38), and protein levels of IκBα and actin were detected by Western blot assay. **B.** Differentiated THP1 cells were pretreated with vehicle (0.5% v/v DMSO) or CRT0066101 (CRT; 10 μM) for 1 hr and then stimulated with medium (med), SR (10 μg/ml), PMA (10 ng/ml) plus Ionomycin (1 nM) or TNFα (25 ng/ml) plus IFNγ (50 ng/ml) for 4 hr. Total RNA was isolated and mRNA levels of the indicated genes were analyzed by RT-PCR.

We investigated whether *S. rectivirgula* could induce activation of PKDs in human macrophages, whether PKD activation by *S. rectivirgula* is also dependent on the kinase function of IRAK4 (a key proximal kinase downstream of MyD88 in the TLR signaling pathway), and whether PKD plays a biological role in the inflammatory response to *S. rectivirgula* in human macrophages using PKD-specific inhibitor CRT0066101. Panel A shows that *S. rectivirgula* induced phosphorylation of PKDs (an indication of PKD activation) and MAPKs (JNK, ERK, and p38), and degradation of IκBα (an indication of NF-κB activation) in THP1 cells. In contrast, phosphorylation of PKDs and MAPKs, and degradation of IκBα in response to *S. rectivirgula* were substantially inhibited in THP1 cells that were pretreated with an IRAK4 kinase inhibitor (IRAK inhibitor 1) or PKD inhibitor CRT0066101. Of note, IRAK inhibitor 1 did not suppress activation of PKD by PMA in THP1 cells, indicating that IRAK inhibitor 1 is specific and not toxic to THP1 cells at the concentration we used in our study. Panel B shows that *S. rectivirgula* induced expression of proinflammatory cytokine and chemokine TNFα, IL-6, and CXCL8 in THP1 cells and that CRT0066101 pretreatment substantially inhibited *S. rectivirgula*-mediated expression of TNFα, IL-6, and CXCL8 in THP1 cells. The specificity of CRT0066101 for PKD and lack of toxicity to THP1 cells (at the concentrations used) has been reported previously (56). Taken together, our results demonstrate that as in murine macrophages,

S. rectivirgula induces PKD activation via an IRAK4-dependent pathway and that PKDs play a critical role in the *S. rectivirgula*-induced inflammatory responses in human macrophages.
